# Oocyte Polarization Is Coupled to the Chromosomal Bouquet, a Conserved Polarized Nuclear Configuration in Meiosis

**DOI:** 10.1371/journal.pbio.1002335

**Published:** 2016-01-07

**Authors:** Yaniv M. Elkouby, Allison Jamieson-Lucy, Mary C. Mullins

**Affiliations:** Department of Cell and Developmental Biology, Perelman School of Medicine, University of Pennsylvania, Philadelphia, Pennsylvania, United States of America; University of Copenhagen, DENMARK

## Abstract

The source of symmetry breaking in vertebrate oocytes is unknown. Animal—vegetal oocyte polarity is established by the Balbiani body (Bb), a conserved structure found in all animals examined that contains an aggregate of specific mRNAs, proteins, and organelles. The Bb specifies the oocyte vegetal pole, which is key to forming the embryonic body axes as well as the germline in most vertebrates. How Bb formation is regulated and how its asymmetric position is established are unknown. Using quantitative image analysis, we trace oocyte symmetry breaking in zebrafish to a nuclear asymmetry at the onset of meiosis called the chromosomal bouquet. The bouquet is a universal feature of meiosis where all telomeres cluster to one pole on the nuclear envelope, facilitating chromosomal pairing and meiotic recombination. We show that Bb precursor components first localize with the centrosome to the cytoplasm adjacent to the telomere cluster of the bouquet. They then aggregate around the centrosome in a specialized nuclear cleft that we identified, assembling the early Bb. We show that the bouquet nuclear events and the cytoplasmic Bb precursor localization are mechanistically coordinated by microtubules. Thus the animal—vegetal axis of the oocyte is aligned to the nuclear axis of the bouquet. We further show that the symmetry breaking events lay upstream to the only known regulator of Bb formation, the Bucky ball protein. Our findings link two universal features of oogenesis, the Bb and the chromosomal bouquet, to oocyte polarization. We propose that a meiotic—vegetal center couples meiosis and oocyte patterning. Our findings reveal a novel mode of cellular polarization in meiotic cells whereby cellular and nuclear polarity are aligned. We further reveal that in zygotene nests, intercellular cytoplasmic bridges remain between oocytes and that the position of the cytoplasmic bridge coincides with the location of the centrosome meiotic—vegetal organizing center. These results suggest that centrosome positioning is set by the last mitotic oogonial division plane. Thus, oocytes are polarized in two steps: first, mitotic divisions preset the centrosome with no obvious polarization yet, then the meiotic—vegetal center forms at zygotene bouquet stages, when symmetry is, in effect, broken.

## Abstract 2

This study traces symmetry breaking in zebrafish oocytes to a cellular organizer that controls the configuration of the meiotic polarized chromosomal bouquet, thereby coupling meiosis and oocyte patterning at the nexus of oocyte differentiation.

## Author Summary

In most vertebrates, an early event in egg development involves the establishment of the so-called animal—vegetal axis; this sets up the embryonic body axes and contributes to germ-line specification, and therefore, is key to embryonic development. The animal—vegetal axis is established during oogenesis by the Balbiani body (Bb), an aggregate of specific mRNAs, proteins, and mitochondria, which forms adjacent to the nucleus and ultimately defines one pole of the oocyte, the vegetal pole. Despite its universal conservation, how the Bb forms and how its position is determined is unknown. Here, we show that Bb formation is initiated at the onset of meiosis, and its position coincides with a previously known meiotic polarized nuclear configuration, the chromosomal bouquet, which gathers the chromosome ends, the telomeres, asymmetrically on the nuclear membrane to assist in homologous chromosome pairing. We reveal that a global cellular organizer functioning via microtubules generates the bouquet and aggregates the Bb precursors asymmetrically towards the centrosome. We determined that these events lie functionally upstream to the Bb regulator Bucky ball. Further upstream, we found that the centrosome appears prepositioned by an intercellular cytoplasmic bridge derived from the last presumptive cell division plane of the premeiotic oogonial cell. Thus, oocyte polarity and the chromosomal bouquet are linked through a common cellular polarization mechanism.

## Introduction

Cell polarity is essential to epithelial tissue formation and function in both development and homeostasis. Correct cellular polarization is required for proper asymmetric cell division of stem cells, as well as the appropriate segregation of cell fate determinants to daughter cells in the generation and maintenance of functioning tissues [1–3]. Aberrant polarization in stem cells, their daughter cells, and differentiated cells causes tissue defects and cancer [3,4]. Tracing the origins of cell polarity in many systems has, therefore, been of great biological and clinical interest.

In most vertebrates, oocyte polarization along the animal—vegetal (AV) axis is key to establishing the embryonic body axes, as well as specifying the germline. First, the embryonic dorsoventral axis is established by dorsal determinants localized to the egg vegetal pole during oogenesis. Following fertilization, these vegetally-localized dorsal determinants then translocate via the Syntabulin linker, Kinesin motor, and cortical microtubules to the future dorsal side of the embryo, where they activate a Wnt signaling pathway [5–11]. Nuclear localization of β-catenin then establishes the dorsal organizer, generating the embryonic dorsoventral axis [8,12]. Importantly, the mRNAs of *syntabulin*, as well as of dorsal determinants like *wnt11* and *wnt8* in Xenopus and zebrafish, localize to the oocyte vegetal pole [7,9–11]. Disrupting their localization and/or function results in ventralized embryos that lack dorsal structures [7–9,11,12].

Secondly, germ cells form by inheriting vegetally-localized germ cell determinants, termed germ plasm, during cleavage stages. The germ plasm initially adheres to the cleavage furrows and then segregates to daughter cells during asymmetric cell divisions, inducing germline fate in specific blastomeres. Importantly, prior to localization to furrows, the germ plasm components are aggregated at the egg and oocyte vegetal pole [8,9,13–19]. Finally, the AV axis of the oocyte aligns with and determines the embryonic anterior—posterior axis. Vegetal pole formation in the oocyte thus provides crucial positional information to the future developing embryo.

The oocyte vegetal pole is specified by the Balbiani body (Bb, also called the “mitochondrial cloud” in Xenopus). The Bb is an aggregate of mRNA protein granules (mRNP) that include embryonic patterning factors and germ plasm, as well as organelles such as mitochondria [16,17,20]. Following Bb formation adjacent to the nucleus, it associates with the oocyte cortex, where it unloads its mRNPs to specify the vegetal pole of the oocyte [8,14–16,21,22]. Demonstrating the developmental importance of the Bb, most of the dorsal determinants and germ plasm components discussed above localize to the Bb and then the vegetal pole. Moreover, the initial Bb pathway is likely further required for a second wave of vegetal mRNA localization in oogenesis, termed the “late pathway”, which utilizes a specific microtubule population and Kinesin motors to localize additional factors to the vegetal pole [23,24]. Loss of the Bb impedes mRNA localization by this pathway as well [21]. Despite the importance and universal conservation of the Bb from insects to mammals [16,20,25,26], its formation is poorly understood. In particular, it is unknown whether its cellular position is determined stochastically or by a prepattern. The source of oocyte AV polarization is, therefore, unknown.

Since the Bb forms intimately associated with the nucleus, we postulated that the nucleus may determine the position of the Bb. Such a mechanism would require polarized cues from the nucleus. Intriguingly, a polarized nuclear configuration, called the chromosomal bouquet, arises preceding Bb formation. During the zygotene stage of meiosis I, the chromosomes orient as a bouquet with their telomeres clustered to one pole of the nuclear envelope (NE), while the chromosome loops face the opposing side [27]. The bouquet is a universal feature of meiosis and promotes the synapsis of homologous chromosomes to facilitate recombination [27–33]. We hypothesized that the bouquet plays an additional role in oocyte polarity, whereby the nuclear axis of the bouquet predicts the oocyte AV axis.

Using quantitative image analysis and markers to define the early meiotic oocyte stages in zebrafish, we characterized the previously inaccessible early meiotic stages and analyzed the chromosomal bouquet configuration in zebrafish. We traced the first asymmetric localization of Bb precursor components to the telomere cluster of the bouquet as early as the onset of meiosis. We show that these early symmetry-breaking events lay upstream to the function of Bucky ball (Buc), the only known regulator of Bb formation. We further show in vivo that microtubules are required for both telomere clustering and Bb precursor localization during the zygotene bouquet, indicating that these cytoplasmic and nuclear events are mechanistically coordinated. In addition, we found that meiotic oocytes in zebrafish develop in nests with specialized cytoskeletal features, revealing a higher order organization of the nest. This organization indicates that centrosome positioning in meiotic oocytes is predetermined by a previous mitotic division. Overall, our data show that oocytes are polarized in two steps: first, mitotic divisions preset centrosome localization, but no other asymmetry is apparent; then, in the zygotene stage, symmetry is broken in a microtubule-dependent manner that clusters the telomeres of the bouquet and localizes Bb precursors to the region of the centrosome. To our knowledge, this is the first dissection of the early dynamics of oocyte symmetry breaking in a vertebrate, revealing that oocyte polarity aligns with a conserved nuclear polarity.

## Results

### Bb Precursor Components Collectively Polarize in a Nuclear Cleft

**Fig 1:**
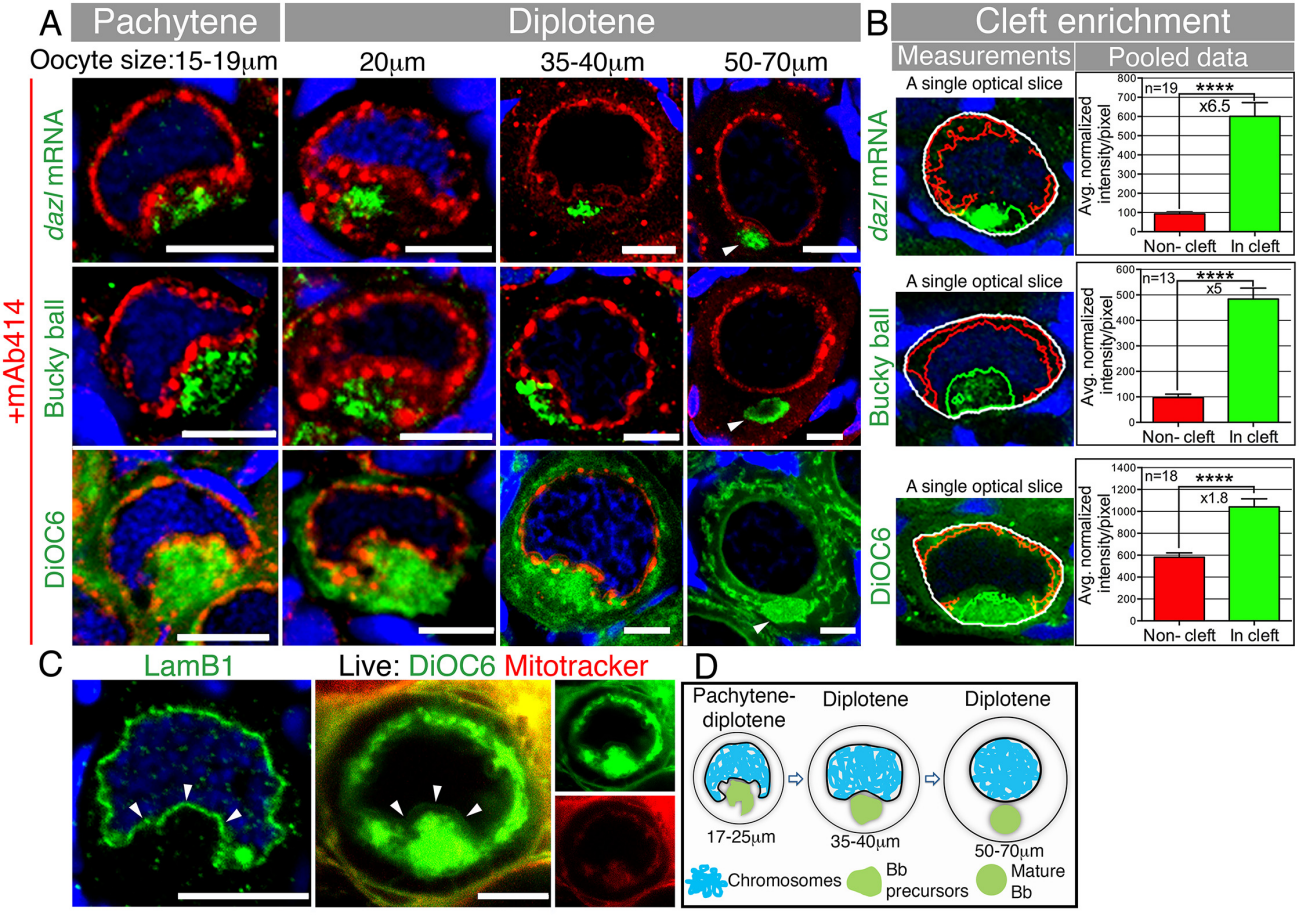
The Bb begins to form in a nuclear cleft at the onset of pachytene. **(A)** Tracking of *dazl* mRNA, Buc, and DiOC6 (membrane marker of organelles), together with mAb414 (a marker of nuclear pores, red) and DAPI (blue). The nuclear cleft morphology and in-cleft aggregation at pachytene through diplotene (≤40 μm) stages, as well as the normal Bb at diplotene (50–70 μm) stages, were observed in 100% of the oocytes (*dazl*, *n* = 26 ovaries; Buc, *n* = 8 ovaries, DiOC6, *n* > 100 ovaries). mAb414 detects the NE (fine line) and perinuclear granules (spherules, S4C Fig). mAb414 channel is omitted in DiOC6 50–70 μm. Arrowheads: mature Bb. In all figures, images are partial sum projections, unless noted otherwise. Scale bar: 10 μm. Oocyte sizes (diameter in μm) are indicated at top of panels. **(B)** Quantification of Bb precursor enrichment in the nuclear cleft of pachytene to early diplotene oocytes (17–25 μm). Left panels are examples of automated measurements (cleft analysis; white, entire measured cytoplasm; red, noncleft cytoplasm; green, cleft cytoplasm; S1 Fig shows the full stacks; S1 Data). Mean and standard error of the mean (SEM) of pooled data are plotted in the graphs. *p*-value, ****<0.0001; average fold enrichment is indicated (×). **(C)** LamB1 and DAPI (blue) labeling in fixed ovaries (*n* = 15 ovaries), and images of live oocytes from whole ovaries (*n* = 13 ovaries) stained with DiOC6 and Mitotracker, confirms the shape of the NE (arrowheads) during cleft stages. **(D)** A schematic of the Bb precursor aggregate during cleft stages and in the mature Bb.

To address when oocyte polarity is generated, we examined the localization of three core Bb components at earlier stages than previously studied: *dazl* mRNA, Buc, and the typical Bb-concentrated mitochondria (the Bb is also called the “mitochondrial cloud” in Xenopus) [21]. As previously shown [21,34,35], all three components localize to the mature Bb at the nuclear periphery of mid-diplotene stages (Fig 1A; oocyte size of 50–70 μm in diameter). In earlier diplotene stages, we detected the three Bb components aggregated within an indentation of the NE (Fig 1A; 35–40 μm oocyte). Even earlier, at the onset of the diplotene stage, the nucleus exhibited a unique highly concave face, forming what we term a “nuclear cleft” (Fig 1A; 20 μm oocyte; S1 Video). At this stage all three Bb components were aggregated in the cytoplasm within the nuclear cleft (Fig 1A), which was also evident even earlier at the pachytene stage (Fig 1A; 15–19 μm oocyte). We developed an algorithm to measure the Bb precursor intensities in the cleft versus noncleft cytoplasm of pachytene to early diplotene oocytes (“cleft analysis”, Materials and Methods). Our cleft analysis shows a robust enrichment of *dazl* (×6.5), Buc (×5.0), and DiOC6 (×1.8) in the cytoplasm of the nuclear cleft (Fig 1B; S1–S3 Figs, S1 Data). At all stages shown, mAb414 detected both the NE (fine line) and perinuclear granules (spherules).

The concave shape of the NE during pachytene to diplotene stages was confirmed by LaminB1 labeling, images of live oocytes labeled with vital dyes, and ultrastructural analysis of whole ovaries (Fig 1C, S2 Fig, S1–S3 Videos). We found that the nuclear cleft was most pronounced at the onset of diplotene and then gradually recedes to a typical spherical nucleus as the mature, spherical Bb forms (Fig 1D). Interestingly, cleft stage nuclei lack detectable A-type lamins (LamA/C) that are evident at postcleft stages (S2 Fig, S4 Video). Nuclear lamin composition controls the rigidity of the nucleus [36,37]. The specific absence of LamA/C from cleft stages may provide the nuclear flexibility that allows the cleft to form and suggests that this transient nuclear morphology is developmentally regulated.

### Characterization of Early Meiosis in Zebrafish Oocytes

We found that Bb precursor components are polarized in an aggregate within a nuclear cleft at the onset of pachytene much earlier than previously known. We thus investigated if Bb precursors aggregate earlier in meiosis. However, early stages of zebrafish oogenesis are not well characterized, and meiosis was not addressed [38–40]. We therefore characterized the stages preceding pachytene, the leptotene and zygotene bouquet stages. We investigated the telomere dynamics characteristic of these early meiotic stages, which is conserved across species [27], using a telomere FISH marker.

**Fig 2:**
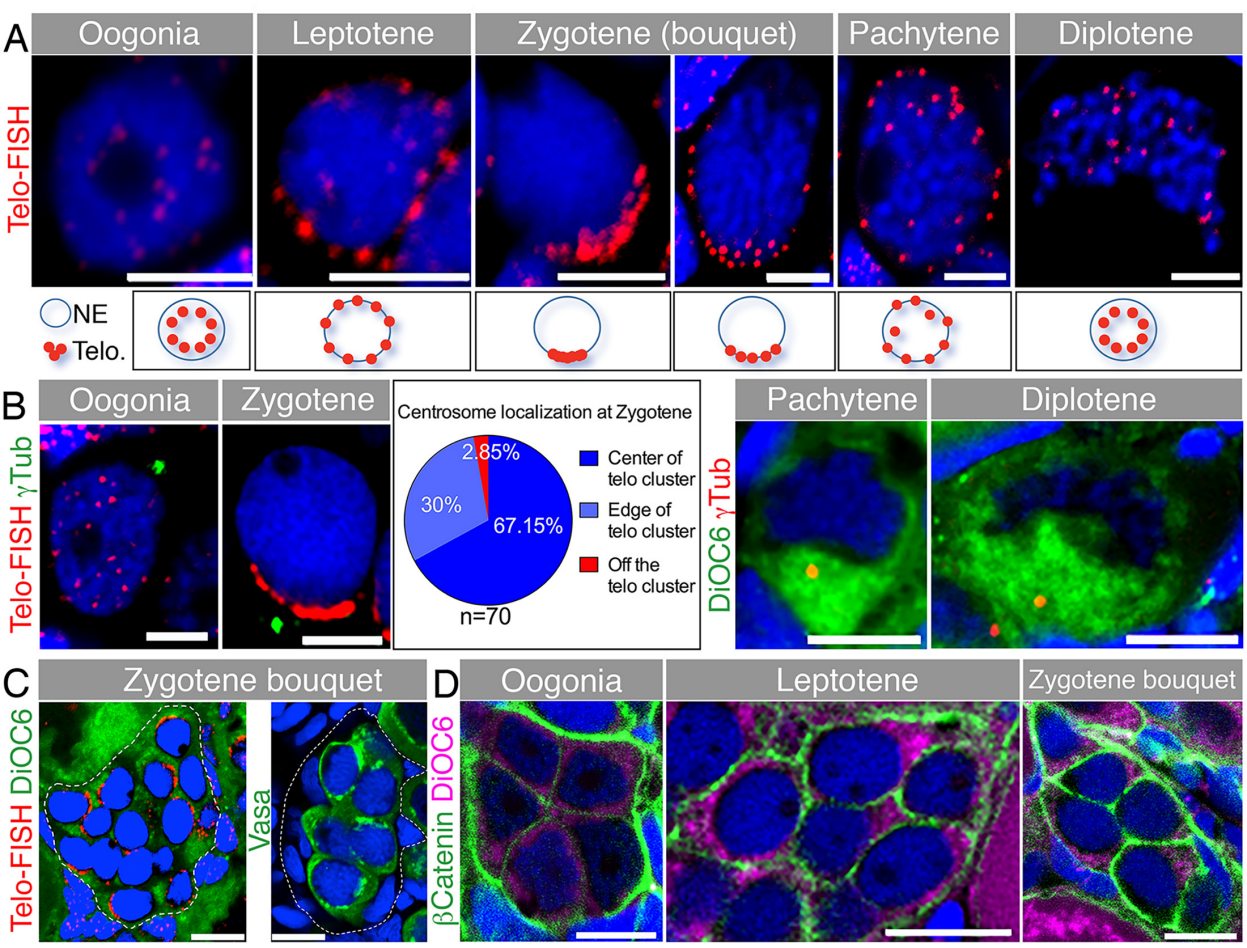
Telomere dynamics and the centrosome during early meiosis and the chromosomal bouquet configuration. **(A)** Telomere (Telo-FISH) dynamics in early meiotic zebrafish oocytes costained with DAPI (blue). Nuclear zoom-in views are shown (*n* = 41 ovaries). Scale bar: 5 μm. Lower panels show schematics of telomere positions in the corresponding stages. **(B)** Centrosome localization during early meiosis. Left: costaining of γTub, Telo-FISH and DAPI (blue; *n* = 8 ovaries). Pie chart: frequencies of zygotene centrosome localization relative to the telomere cluster. Right: costaining of γTub, DiOC6 and DAPI (blue; *n* = 7 ovaries). Scale bar: oogonia, zygotene is 5 μm; pachytene, diplotene is 10 μm. **(C)** Development of early meiotic oocytes in nests. Left: A group of zygotene oocytes (clustered telomeres marked by Telo-FISH in red) reside in a cluster (outlined) with follicle cells (amorphous smaller DAPI-positive cells) not apparent between them, but are present in the cluster periphery (*n* = 41 ovaries). Right: Detection of the germ cell specific marker Vasa (green) in zygotene cells (staged according to A—B and S3 Fig) are surrounded by Vasa-negative, DAPI-positive (blue) somatic follicle cells. The nest is outlined (*n* = 11 ovaries). Scale bars: 10 μm. Video S7 shows a 3-D view of a nest, demonstrating the intimate clustering of the zygotene oocytes, surrounded by follicle cell nuclei. **(D)** Early oocytes in the nest show adjacent cytoplasmic membranes (β-Catenin; green), costained with DiOC6 (magenta) and DAPI (blue) (*n* = 22 ovaries). Scale bar: 10 μm.

We also examined centrosome localization, which functions in mouse spermatocytes to cluster the telomeres of the bouquet stage (Fig 2, S3 Fig) [41]. We found that in premeiotic oogonia, telomeres are randomly distributed in nuclei but become loaded radially on the NE at the leptotene stage (Fig 2A); the centrosome at these stages is positioned perinuclearly (Fig 2B). During zygotene stages, telomeres are tightly clustered on the NE, forming the bouquet configuration (Fig 2A, S5 and S6 Videos).

We found that the centrosome localized to the cytoplasm facing the telomere cluster at this stage (Fig 2B). At the pachytene stage, telomeres redistributed radially on the NE then detached from the NE at the onset of diplotene (Fig 2A). At these two stages, the centrosome localized roughly to the center of the clearly evident nuclear cleft (Fig 2B). At later diplotene stages (>25 μm), we could not detect the centrosome, consistent with its known loss during oogenesis [42,43].

During our analysis, we found that early meiotic zebrafish oocytes develop in nests (Fig 2C). A germline cyst is defined as a cluster of germ cells, which are connected via cytoplasmic bridges that remain from previous incomplete mitotic divisions and are collectively surrounded by somatic follicle cells. A nest defines the same organization as that of the cyst, but where cytoplasmic bridges were not directly demonstrated. Development of germ cells within cysts is conserved from insects to mammals [44]. However, in zebrafish only premeiotic oogonia, not meiotic oocytes, were described in nests [21,38,40]. We observed zygotene bouquet stage cells tightly adjacent to each other, with no follicle cells apparent between them, although they were detectable around the zygotene cell groups (Fig 2C, S7 Video). β-Catenin staining of cell membranes was similar in premeiotic oogonia, leptotene, and zygotene cells consistent with these cells residing within a nest (Fig 2D). Electron micrograph (EM) images confirmed the presence of only two membranes, tightly juxtaposed to each other, between zygotene oocytes, ruling out the presence of follicle cells between oocytes (S4A Fig, red arrowheads; *n* = 19 oocytes in 7 nests of two ovaries). While this organization may represent a cyst, we did not directly examine cytoplasmic bridges between oocytes and therefore define these as meiotic nests.

### Earliest Asymmetry at Zygotene Bouquet Stage

**Fig 3:**
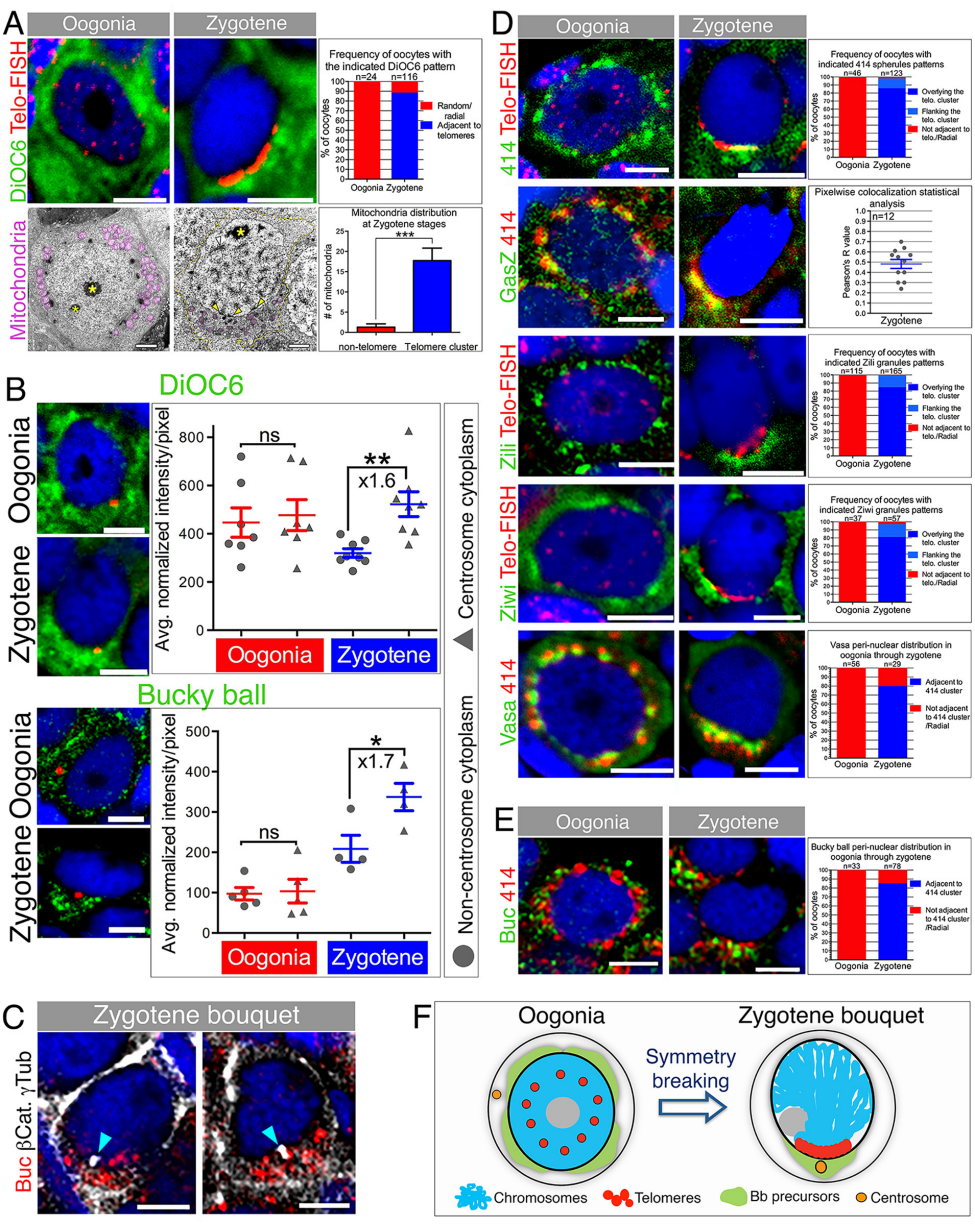
Aggregation of Bb precursor components in the telomere cluster cytoplasm. **(A)** Mitochondria localize to the telomere cluster. Top: DiOC6 pattern relative to telomeres (Telo-FISH) in oogonia and zygotene (DAPI, blue). Scale bar, 5 μm. Graph: DiOC6 pattern frequencies (S1 Data). Bottom: mitochondria (pseudocolored magenta) in oogonia and zygotene and relative to the presumptive telomere cluster. At zygotene, SCs are detected (arrowheads). SC-NE contact points mark the presumptive telomere cluster (yellow arrowheads). The cell membrane is outlined (orange) in zygotene. *, nucleoli. Scale bar, 2 μm. Graph: counts of mitochondria around the zygotene nucleus (*n* = 7 oocytes; S4 Fig; S1 Data). **(B)** DiOC6 and Buc distribution in oogonia and zygotene. Zygotene nest analysis (Materials and Methods) results are plotted showing enrichment around the centrosome cytoplasm (triangles) versus the remaining cytoplasm (circles) in zygotene but not oogonia (DiOC6: 6 ovaries ‒ Zygotene, *n* = 168 oocytes in 8 nests, Oogonia, *n* = 59 oocytes in 7 nests; Buc: 10 ovaries − Zygotene, *n* = 111 oocytes in 4 nests, Oogonia, *n* = 21 oocytes in 5 nests). *p*-value: *<0.05; **<0.01; ns: not significant. Bars indicate mean and SEM. Average fold enrichment is indicated (×). Data in S1 Data. Scale bar, 5 μm. **(C)** Buc localizes to the centrosome (white) in zygotene (*n* = 13 ovaries). Labeling cell membranes (β-catenin; white) and analyzing individual oocytes confirms Buc localization to the centrosome cytoplasmic region (cyan arrowheads; 100%, *n* = 14 oocytes). Two examples are shown. Scale bar, 5 μm **(D)** GasZ and piRNA pathway granules localize to the telomere cluster. mAb414 spherules, Zili and Ziwi patterns relative to telomeres (Telo-FISH), and GasZ and Vasa relative to mAb414 in oogonia and zygotene (DAPI costained, blue). Scale bar, 5 μm. mAb414, Zili, Ziwi, and Vasa graphs indicate patterns frequencies (S1 Data). GasZ graph shows pixel-wise Pearson correlation coefficient of GasZ and mAb414 signals, confirming colocalization (Materials and Methods; S1 Data). **(E)** mAb414 NE spherules (red) position in oogonia and zygotene oocytes further confirms Buc (green) radial distribution in oogonia and localization to the telomere cluster apposing cytoplasm at zygotene in individual cells. Graph indicates patterns and frequencies (S1 Data). **(F)** A schematic of oocyte symmetry breaking at the zygotene bouquet.

Having identified the oogonia, leptotene, and zygotene stages, we then examined Bb precursor components during these oogenesis stages. We first addressed mitochondrial aggregation with DiOC6 labeling, which detects the Bb-enriched mitochondria (Fig 1A–1C). While the DiOC6 signal was radially distributed in premeiotic oogonia, it was enriched in zygotene stages in the perinuclear cytoplasm apposing the telomere cluster (“telomere cluster cytoplasm”; Fig 3A, S1 Data). Since DiOC6 does not detect mitochondria specifically, we examined mitochondrial localization ultrastructurally. Transmission electron microscopy (TEM) images clearly showed radially distributed mitochondria in oogonia (Fig 3A). At zygotene stages, the contact of synapsed chromosomes (SCs) with the NE marks the presumptive telomere cluster (Fig 3A, arrowheads), consistent with our zygotene confocal observations (Fig 3A, top). Counting the number of mitochondria in high-power images of the entire cytoplasm 360° around the nucleus (S4A Fig) confirms the enrichment of mitochondria to the cytoplasm apposing the presumptive telomere cluster (Fig 3A, S1 Data). We conclude that mitochondria are first aggregated in the telomere cluster cytoplasm at zygotene.

To measure potential enrichment of Bb precursor components in the telomere cluster cytoplasm, we developed a quantitative, high-throughput MATLAB program we term “zygotene nest analysis” (Materials and Methods). This algorithm is based on our observation that the centrosome is a reliable marker of the telomere cluster cytoplasm (Fig 2B) and tests for enrichment of other components in the cytoplasm surrounding it. We first measured the enrichment of DiOC6 labeling at the zygotene bouquet stage (Fig 3A). Indeed, our nest analysis results showed a 1.6-fold DiOC6 enrichment to the telomere cluster cytoplasm, specifically at zygotene stages (Fig 3B, S1 Data).

We next investigated localization of the Buc protein, the only protein known to be required for Bb formation [21]. Buc localizes to the mature Bb [34] and to the pachytene Bb precursor aggregate (Fig 1A and 1B). We detected Buc radially distributed in oogonia cytoplasm (Fig 3B). However, at zygotene Buc is enriched in the centrosome-telomere cluster cytoplasm (Fig 3B). Nest analysis reveals a 1.7-fold enrichment versus the remaining cytoplasm (Fig 3B, S1 Data), specifically at zygotene. As a control, we measured intensities of a transgene-driving DCLK-GFP (*Tg(Ef1α*:*Dclk-GFP)*), a microtubule binding protein [6]. Nest analysis showed no specific enrichment in either oogonia or zygotene oocytes (S4B Fig, S1 Data). We investigated Buc localization relative to the centrosome in zygotene oocytes, colabeling cell membranes with β-Catenin. Indeed, Buc localized to the centrosome cytoplasmic region of zygotene oocytes (100%, *n* = 14 oocytes; Fig 3C). Therefore, Bb precursor components become specifically enriched in the telomere cluster cytoplasm at the zygotene stage.

GasZ is another protein resident to the Bb in mid-to-late diplotene stages [21,45] (S4D Fig). GasZ serves as a scaffold protein for the piRNA machinery in perinuclear nuage in mice testis [46]. We found that perinuclear granules in early zebrafish oocytes contain the Vasa, Ziwi, and Zili proteins colocalized with the nuclear pore complex marker mAb414 (perinuclear spherules; S4C Fig), similar to Vasa (GLH-1) P granule localization to nuclear pores in *Caenorhabditis elegans* [47,48]. Consistent with its localization in mice testes, we detected GasZ colocalized with mAb414 to these perinuclear granules radially around oogonia nuclei (Fig 3D, S4D Fig). GasZ immunostaining was not compatible with the Telo-FISH protocol, preventing a direct test for colocalization in the bouquet. However, we found that mAb414 spherule immunostaining localized to the telomere cluster at the bouquet stage (Fig 3D). We therefore used mAb414 spherules as a reference for GasZ localization in the bouquet. GasZ exhibited a polarized pattern in the bouquet, similar to that of mAb414 spherules (Fig 3D). Pixel-wise statistical analysis in zygotene stages confirmed colocalization of GasZ and mAb414 signals (average Pearson correlation coefficient = 0.5; Fig 3D, S1 Data; ~75% of each signal was colocalized with the other, Manders coefficient). GasZ bouquet localization was also confirmed by costaining for GasZ and DiOC6. Therefore, the Bb precursor GasZ protein localizes to the telomere cluster cytoplasm of the bouquet.

Consistent with GasZ and the mAb414 spherule dynamics, the piRNA processing enzymes, Ziwi and Zili, and the RNA binding protein in the piRNA pathway Vasa, exhibited the same transition from radial granules in oogonia to high localization to the telomere cluster apposing cytoplasm (Fig 3D). While a role for such a polarized localization of perinuclear granules in the piRNA pathway is unclear, these granules might have been coopted to contribute components of similar functions, like GasZ, to the forming Bb. GasZ bridges different mRNA binding proteins within the nuage in mice [46,49] and could function similarly within the forming Bb.

Having defined the mAb414 spherules as markers for the telomere cluster position at zygotene (Fig 3D), we utilized it to further analyze individual zygotene oocytes for their Buc localization. We identified zygotene oocytes blind to the Buc channel, and scored oocytes with clear mAb414-clustered localization, typical of its pattern in zygotene bouquet. We then turned on the Buc channel and examined its distribution in reference to the mAb414 signal position. Consistent with our previous analyses, this approach confirmed the transition of Buc from a radial distribution in oogonia to enrichment at the cytoplasm apposing the telomere cluster, as visualized by the mAb414 cluster (Fig 3E, S1 Data).

We conclude that Bb precursors transition from radial distribution in premeiotic oogonia to being enriched in the cytoplasm facing the bouquet telomere cluster (Fig 3F). This association of Bb precursors, the centrosome and the telomere cluster, unveils the first symmetry breaking in the zebrafish oocyte. At this stage, the nuclear axis of the bouquet predicts the cellular AV axis in the oocyte, with the telomere cluster position marking the future vegetal pole.

### The Chromosomal Bouquet Stage Asymmetries Lie Upstream of Buc

**Fig 4:**
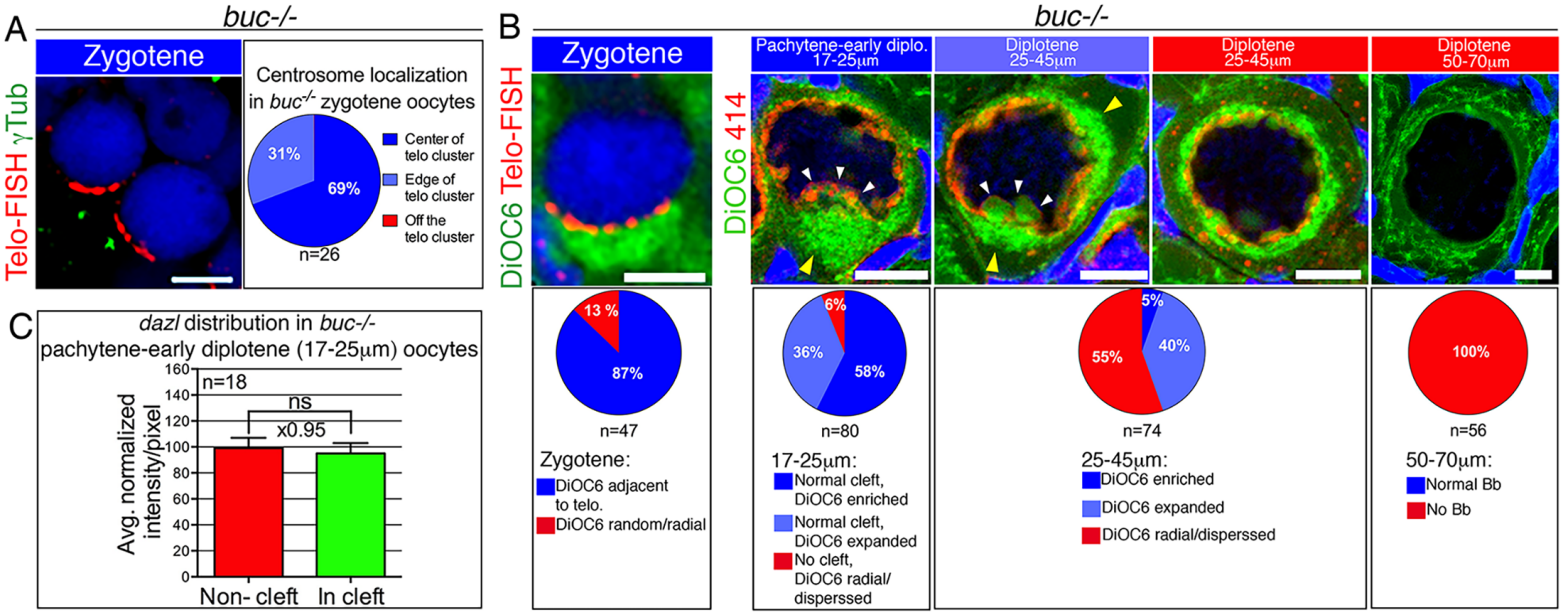
Early oocyte polarization is normal in *buc*^*-/-*^ oocytes. **(A)** Telomere clustering and centrosome positioning (DAPI, blue) are normal in *buc*^*-/-*^ oocytes (*n* = 5 ovaries). Pie chart: centrosome position frequencies. **(B)** *buc*^*-/-*^ ovaries display normal early polarization. Pie charts show the frequencies of each phenotype. The typical phenotype is shown (top), with colors matching the represented phenotype in the charts (*n* = 26 ovaries). White arrowheads, nuclear cleft; yellow arrowheads, DiOC6 enrichment. Scale bars as in Fig 2B. **(C)** Cleft analysis results for *dazl* localization in *buc*^*-/-*^ pachytene to early diplotene oocytes. Compare with Wt in Fig 1B. Mean and SEM are plotted. ns, not significant. Data in S1 Data.

Before our current findings, early steps of Bb formation were unknown. A functional pathway for Bb formation has, therefore, yet to be constructed. We tested the relationship between the zygotene bouquet configuration, localization of Bb precursors in the nuclear cleft, and Buc function, the only known protein required for Bb formation. We found that telomeres in *buc*^*-/-*^ zygotene oocytes cluster normally and the centrosome localizes to the telomere cluster in 100% of the cells similar to wild-type (Wt) (Fig 4A). Concomitant with bouquet formation in *buc*^*-/-*^ oocytes, DiOC6 was also normally enriched in the telomere cluster cytoplasm similar to Wt (Fig 4B). Hence, Buc is dispensable for bouquet formation and early asymmetry formation.

We next examined Bb precursor enrichment in the nuclear cleft stages that follow the bouquet stage in *buc* mutants. Most *buc*^-/-^ oocytes from pachytene through early diplotene (17–25 μm) showed normal morphology, with a nuclear cleft enriched for DiOC6 signal (Fig 4B). Only at late diplotene stages (25–45 μm) were aberrant DiOC6 patterns detected; in most oocytes, DiOC6 was expanded completely radially, while in the remaining oocytes DiOC6 was expanded partly outside the nuclear cleft (Fig 4B). As expected [21], all mid-diplotene *buc* mutant oocytes (≥50 μm) lack a Bb based on both DiOC6 and *dazl* mRNA localization (Fig 4B). These dynamics show that while the mitochondrial aggregate disperses at late diplotene stages, its formation is intact through the zygotene—pachytene stages. Following its normal bouquet localization, the centrosome properly localizes consistently to the nuclear cleft in *buc* mutant oocytes through pachytene to early diplotene stages. Therefore, early polarization appears normal in *buc*^*-/-*^ oocytes. These results altogether demonstrate that Buc is required for Bb formation downstream of the zygotene bouquet configuration and early asymmetry formation, and in parallel to or downstream of nuclear cleft formation.

Interestingly, *dazl* transcript is not enriched in the cleft of *buc*^*-/-*^ pachytene to early diplotene (17–25 μm) oocytes (Fig 4C, S1 Data). We performed quantitative real-time polymerase chain reaction (QRT-PCR) analysis to compare *dazl* levels between Wt and *buc*^*-/-*^ ovaries. No decrease in *dazl* levels was detected, indicating that *dazl* remains present but is no longer localized in *buc*^*-/-*^ oocytes, as reported previously [21,50]. Since Buc does not bind mRNA itself, but does interact with at least one RNA binding protein [34], it likely functions through such interactions to localize *dazl* to the cleft. A role for Buc in actively forming the large RNP Bb granule is consistent with Buc as an intrinsically disordered protein (IDP) [50,51]. IDPs provide adhesive scaffolds in mRNPs, promoting their phase separation from the soluble cytoplasm into distinct bodies [50–54]. Buc might serve a similar adhesive role in the Bb precursor aggregate. In the absence of Buc, the zygotene—pachytene mitochondrial aggregate does form but then disperses and fails to mature into a Bb. While Buc is required for Bb formation, Buc position is set upstream by the centrosome telomere-cluster cytoplasm during the symmetry-breaking events of the zygotene stage.

### Microtubules in Early Meiotic Oocytes

We investigated further the relationship between bouquet formation and early asymmetry of Bb components. During the chromosomal movements that generate the bouquet, telomeres attach to Sun/KASH domain proteins on the NE that connect them with microtubules in the cytoplasm [28,29,31,55]. Importantly, microtubules are required for bouquet formation and synapsis in mice spermatocytes and *C*. *elegans* oocytes [31,41]. Therefore, we characterized microtubule organization during early oogenesis as a possible link between chromosomal bouquet formation and Bb component aggregation.

**Fig 5:**
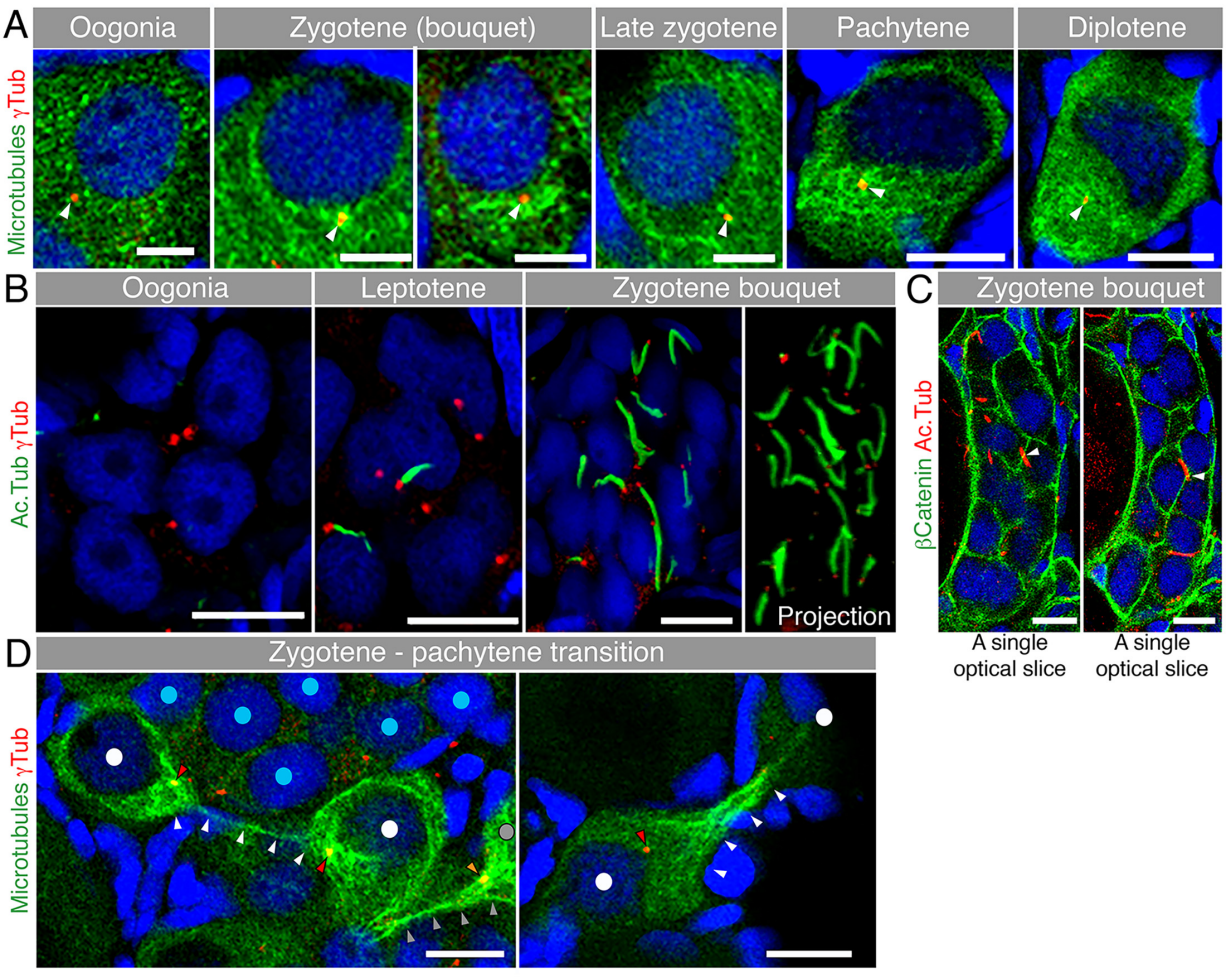
Microtubules in early meiosis. **(A)** Microtubule (*Tg(βAct*:*emtb-3GFP)*, immunostained for GFP, green) organization during early meiotic oocytes costained with γTub (red) and DAPI (blue) (*n* = 6 ovaries). Scale bar: oogonia, zygotene = 5 μm; pachytene, diplotene = 10 μm. **(B)** Acetylated microtubule (Ac.Tub) cables in the nest. Ac.Tub cables (green) associate with centrosomes (red) of leptotene (center) and zygotene (right) oocytes (costained with DAPI), but are not present in oogonia (left) (*n* = 4 ovaries). Scale bar: 10μm. Right panel shows a full projection of Ac.Tub cables and centrosomes in the zygotene nest shown (see also S8 Video). **(C)** Some Ac.Tub cables (red) appeared to extend across the cell membranes (β-Catenin; green) of neighboring oocytes in the nest (costained with DAPI, blue; *n* = 12 ovaries). Arrowheads indicate crossing sites. Scale bar: 10 μm. Two single optical slices of the same nest are shown, see also S9 Video. **(D)** Zygotene to pachytene transitioning oocytes (DAPI, blue) show thin microtubule connections (*Tg(βact*:*emtb-3GFP)*, green; white arrowheads) from the dense microtubule network around the centrosome (red; red arrowheads) of one cell to that of the other (*n* = 6 ovaries). The nuclei of the connected oocytes are labeled with white dots. Grey dot, grey and orange arrowheads indicate another oocyte with its centrosome and microtubule connection to an oocyte outside the image plane. Such oocytes were always observed in the periphery of a zygotene nest (nuclei labeled with blue dots, left panel; outside the image plane of right panel). Scale bar: 10 μm. Arrowheads indicate the centrosomes.

We used a transgenic microtubule reporter line, *EMTB-3GFP* (Fig 5A) [56] and costained for γTubulin to visualize the centrosome. We found rather loose radially symmetric perinuclear microtubules in premeiotic oogonia without significant enrichment around the centrosome (Fig 5A). In zygotene stages, a denser network of microtubules appeared around the centrosome at the cytoplasmic site apposing the telomere cluster. At later zygotene stages the network appeared wider, concomitant with the wider nuclear distribution of telomeres (Figs 5A and 2A). During pachytene and early diplotene stages, a microtubule meshwork was found in the nuclear cleft cytoplasm surrounding the centrosome (Fig 5A). The organization of microtubules around the centrosomes during zygotene suggests that the centrosome functions as a microtubule organizing center (MTOC) at this stage and that the role of microtubules in bouquet formation is conserved in zebrafish.

We also examined acetylated microtubules during these early meiotic stages and found a specialized cytoskeletal organization unique to the meiotic nest. We observed long cables of acetylated tubulin (Fig 5B and 5C). While varying in length, each cable emanated from a centrosome of one cell (Fig 5B, S8 Video). The acetylated cables were specific to leptotene and zygotene nests: they were absent from premeiotic oogonia and were first detected in leptotene oocytes, where they appeared shorter and fewer (Fig 5B), suggesting that they grow and fully mature at zygotene. We never observed the cables in later stage oocytes. Costaining for Acetylated tubulin and telomeres showed that the cables extend from the position of the telomere cluster, consistent with their centrosome association (S6 Fig). Furthermore, costaining with β-Catenin indicates that some of these cables extend between cells in the nest (Fig 5C, S9 Video). This observation also suggests that the cells in the meiotic nest are connected and some of the acetylated cables may extend through cytoplasmic bridges between oocytes.

Interestingly at the zygotene to pachytene transition, we detected microtubules extending significant distances between pairs of cells in what appeared to be cytoplasmic bridges connecting the microtubule meshworks that surround the centrosome of each cell (Fig 5D). We never detected acetylated tubulin staining at the zygotene to pachytene transition when these interoocyte connections were apparent. Only zygotene to pachytene transitioning oocytes displayed these microtubule connections, and they were always located in the periphery of a nest (Fig 5D). These cells may be completing cytokinesis and separating from the nest. Such a release from the nest at the zygotene to pachytene transition is consistent with that of the mouse ovary, where early meiosis occurs in nests and individual oocyte folliculization initiates at pachytene [57].

Intriguingly, the acetylated and then nonacetylated microtubules we observed here strikingly resemble the typical events of late cytokinesis, where midbody microtubules that extend in the cytoplasmic bridges are acetylated but then their deacetylation is required for cytokinesis completion [58–63]. This suggests that zygotene—pachytene-transitioning oocytes complete cytokinesis for their release from the nest. Importantly, the nonacetylated microtubules connect the centrosome regions of the two connected oocytes, indicating that the final mitotic division of oogonia may set the centrosome position at zygotene. Remarkably, the centrosome region at this stage already defines the future vegetal pole of these oocytes. This indicates that the last mitotic division of oogonia may predispose the bouquet configuration and polarization. While polarization is, in effect, established at zygotene, these early events may preset its axis.

### The Bouquet Microtubules Couple Telomere Clustering and Bb Precursor Localization

Our data show that oocyte symmetry breaking can be traced back to the zygotene bouquet stage, when Bb precursors transition from a radial distribution to localization at the centrosome cytoplasm apposing the telomere cluster of the bouquet configuration. We next tested this correlation functionally. Microtubules are key in generating the telomere movements that establish the chromosomal bouquet in mice spermatocytes and in *C*. *elegans* oocytes [31,41]. Our data show that the centrosome—microtubule—telomere bouquet cellular organization found in these organisms is conserved in zebrafish zygotene oocytes. Microtubules are, therefore, strong candidates to link the nuclear telomere movements with the cytoplasmic localization of Bb precursors.

**Fig 6:**
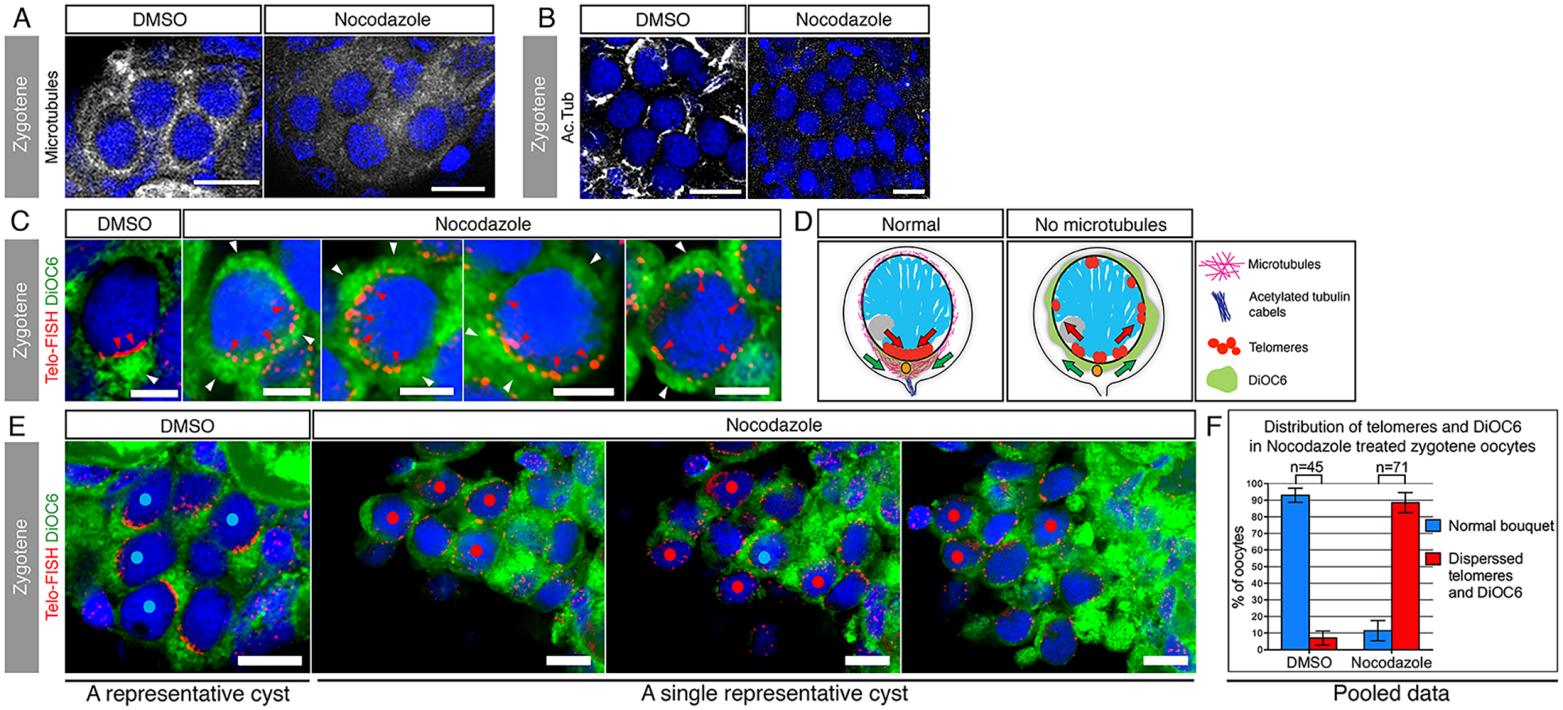
Nuclear telomere clustering and cytoplasmic Bb precursor localization are mechanistically coordinated by the bouquet microtubules. **(A)** Bouquet microtubules (*Tg(βAct*:*emtb-3GFP)*, white) are intact in DMSO-treated ovaries (100%; *n* = 4 ovaries), but depolymerized in nocodazole treated ovaries (100%; *n* = 5 ovaries). A representative zygotene nest is shown (costained with DAPI, blue). Scale bar: 10 μm. **(B)** Acetylated tubulin cables (white) are intact in DMSO ovaries (100%; *n* = 3 ovaries), but are lost in nocodazole treated ovaries (100%; *n* = 6 ovaries; except for rare cases (3 nests) where cables were still intact, all other nest cables were disrupted). A representative zygotene nest is shown (costained with DAPI, blue). Scale bar: 10 μm. **(C)** Microtubules are simultaneously required for telomere clustering and DiOC6 localization at zygotene bouquet. DMSO zygotene oocytes (left panel) show the typical bouquet telomere cluster (Telo-FISH, red, red arrowheads) with the apposing localized DiOC6 (green, white arrowhead), similar to WT control (compare with Fig 4A). In contrast, nocodazole treatments (four right panels) resulted in simultaneous radial expansion of both telomeres and DiOC6. Four representative oocytes are shown. Note the partial to complete radial expansion of telomeres (red arrowheads) and the concomitant expansion and ectopic enrichments of DiOC6 (white arrowheads). Scale bar: 5 μm. **(D)** Schematics of the effects of the loss of microtubules on telomeres and DiOC6 distributions during zygotene bouquet. **(E)** Quantification of the results shown in (C). Zygotene oocytes were scored blind and mid-zygotene oocytes (11.5–13 μm) were selected for analysis. These were examined for their telomere clustering and DiOC6 patterns. A representative DMSO control nest is shown (left), where zygotene oocytes show the typical telomere clustering and apposing DiOC6 localization (nuclei labeled with blue dots). Partial projections of three different planes of a representative nocodazole-treated nest are shown (three right panels sequentially). In this nest, 10 oocytes showed radially-expanded telomeres as well as radial expansion, dispersion, or ectopic enrichments of DiOC6 (nuclei labeled with red dots), and one oocyte was normal (blue dot). Scale bar: 10 μm. **(F)** Pooled statistics of the analyzed nests as in (E). 93% (*n* = 45) of DMSO midzygotene oocytes were normal, while only 11.5% of midzygotene oocytes in nocodazole-treated ovaries were normal and 88.5% (*n* = 71) showed the effects described in (C, E). Bars are standard deviation (SD) of two independent experiments. Data in S1 Data.

To examine microtubule function and test the functional correlation of Bb precursor localization and telomere clustering, we aimed to disrupt microtubules and monitor Bb precursors and telomeres simultaneously. We established a protocol to isolate whole ovaries, culture them for a short period, and treat them for 80 min with the microtubule depolymerizing drug nocodazole or dimethyl sulfoxide (DMSO) as a control. The two ovaries from each fish were split between the nocodazole and DMSO groups, with the latter serving as an internal control. Nocodazole depolymerized microtubules of all oocyte stages, including zygotene bouquet oocytes (Fig 6A). Unexpectedly, we also found that nocodazole disrupted most of the acetylated tubulin cables of zygotene oocytes (Fig 6B).

We then examined Bb precursors and telomeres in zygotene oocytes where both populations of microtubules are absent. To monitor Bb precursors we used DiOC6 to mark Bb mitochondria, since it is the best Bb precursor marker that is compatible with the telomere FISH staining (the FISH protocol requires harsh conditions, which are deleterious for most antibodies and epitopes). We found that in control DMSO-treated ovaries, zygotene oocytes showed normal clustering of telomeres and typical localization of DiOC6 in the apposing cytoplasm (Fig 6C). In contrast, nocodazole-treated zygotene oocytes showed partial to complete radial dispersion of telomeres. Moreover, the same oocytes showed a concomitant expansion of DiOC6 (Fig 6C). We never observed an oocyte where one but not the other was affected. Fig 6D schematizes these effects.

We next quantified these results. To avoid misinterpretation of results, we avoided early and late zygotene stage oocytes where telomere clustering is still incomplete or starting to disperse, respectively. Midzygotene oocytes display condensed chromosomes, a single peripheral nucleolus, and are approximately 12 μm in diameter, (10–11 μm are early and 14–15 μm are late zygotene). To test our ability to identify midzygotene oocytes based on these criteria, we scored the cells blind to the telomere channel and only selected oocytes of 11.5–13 μm in diameter. We then turned on the telomere channel and analyzed telomere clustering and DiOC6 patterns. Using this method, we found that 93% of DMSO-treated oocytes showed normal telomere clustering and apposing DiOC6 localization (Fig 6E, blue dots, *n* = 45 oocytes in 7 nests of 2 ovaries). However, following nocodazole treatment, 88.5% of oocytes showed broad expansion of both telomeres and DiOC6, as in Fig 6C and 6E (Fig 6E red dots, *n* = 71 oocytes in 11 nests of 4 ovaries). Fig 6F shows the pooled results (S1 Data). These results demonstrate that microtubules are required for both telomere clustering and DiOC6 localization during zygotene bouquet, strongly arguing that they are coregulated and functionally linked.

In our experimental settings, we disrupted both the dynamic microtubule population and the acetylated tubulin cables. We cannot distinguish if one or both populations are required for telomere clustering and DiOC6 localization. However, we observed cases where nests in nocodazole-treated ovaries still showed intact acetylated tubulin cables, but DiOC6 was still dispersed. Since perinuclear microtubules were always disrupted, we believe that they may be more directly involved in telomere clustering and DiOC6 localization, and while the acetylated cables may be required, they are not sufficient.

## Discussion

**Fig 7:**
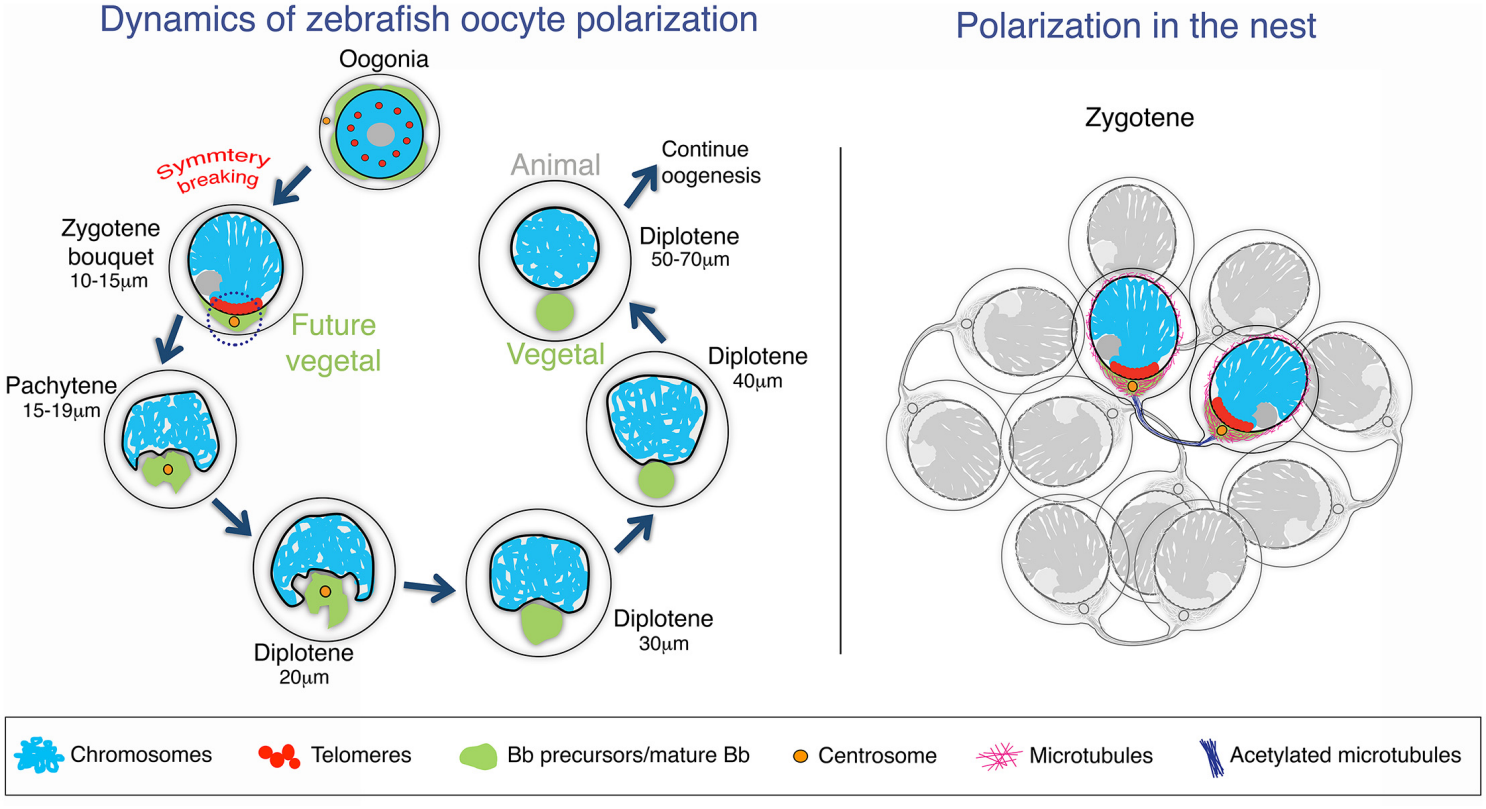
Dynamics of zebrafish oocyte polarization. **(A)** Oocyte symmetry breaking model and early Bb formation. In the premeiotic oogonia, Bb precursors are distributed radially in the cytoplasm, telomeres are scattered intranuclearly, and the centrosome is at a perinuclear position. Nuclear and cytoplasmic asymmetry is first evident when meiosis initiates, and the centrosome and the telomere cluster form the meiotic—vegetal center (circled by a dashed line) during zygotene stages. Bb precursors transition to the cytoplasm localized around the centrosome and adjacent to the telomere cluster of the zygotene bouquet configuration. At this stage the nuclear axis of the bouquet configuration predicts the animal—vegetal cellular axis of the oocyte, with the telomere cluster and the centrosome marking the future vegetal pole. At the subsequent pachytene stage, the nuclear cleft forms at the position of the centrosome where the Bb precursor components also aggregate. The nuclear cleft is most pronounced at the onset of diplotene (20 μm). Throughout pachytene and early diplotene (17–25 μm), the centrosome is found at the center of the Bb precursor aggregate and the cleft. During the following diplotene stages, the nuclear cleft gradually rounds out and the mature, spherical Bb forms, marking the definitive vegetal pole in the oocyte. **(B)** Model for polarization in the cyst. Oogonia in the cyst are connected by cytoplasmic bridges due to incomplete cytokinesis. With the onset of meiosis, specialized acetylated tubulin cables may connect sister oocyte pairs through the cytoplasmic bridges of the last mitotic oogonial division (a highlighted pair is shown). The division plane of the mitotic oogonia therefore positions the centrosome and the future meiotic—vegetal center at zygotene. While the polarity axis may be preset in the mitotic oogonia, polarization is only active at the onset of meiosis, when symmetry is in effect broken.

We trace the first asymmetry in the zebrafish oocyte to chromosomal bouquet formation at the onset of meiosis, much earlier than previously known. Asymmetry is established when Bb precursors transition from radial distribution in premeiotic oogonia to being enriched in the cytoplasm facing the bouquet telomere cluster. Telomere clustering in the bouquet promotes homologous chromosomes to pair, facilitating recombination [28–33,64]. We now report that Bb precursor components localize to the associated centrosome and telomere-cluster cytoplasm of the bouquet, facilitating Bb formation and vegetal pole specification (Fig 7A). To our knowledge, our finding that the bouquet nuclear axis aligns with the oocyte AV axis provides the first evidence for the bouquet being linked to a process outside of meiosis. We propose that the bouquet association of the centrosome and the telomere cluster comprise a meiotic—vegetal center that couples meiotic genetic events with oocyte patterning.

The chromosomal bouquet is a universally conserved meiotic feature discovered in 1900 [27], and the Bb is a universally conserved oocyte feature discovered in 1845 [20]. For the first time to our knowledge, we link these two fundamental oocyte features in the establishment of oocyte polarity. In most vertebrates, oocyte polarization is crucial in setting up the body axes and specifying cell fates in embryonic development [8,9,13–17]. In Xenopus, where the mature Bb has been well described, early dynamics of Bb precursors is lacking. While mitochondrial aggregates were described in premeiotic oogonia, it is not clear whether these are directly related to the mitochondria aggregates within the later mature diplotene Bb [14]. The potential roles of the mammalian Bb and oocyte polarity in general are less clear. Nonetheless, a Bb does form in oocytes of all mammals including human [16,26], comprising at least a transient polarized oocyte morphology. While the mouse Bb is detected at diplotene stages between P3–P7, localization of Bb precursors at zygotene stages (E14.5) was not addressed [26]. The wide conservation of the bouquet and the Bb argues for the conservation of the meiotic—vegetal center in generating polarity generally in oocytes.

To our knowledge, we provide the first evidence that a polarized nuclear morphology predicts cellular polarity. During asymmetric cell division in mitotic cells, interactions between the centrosome and the chromosomes or the nucleus serve as a platform for a polarized segregation of cell fate determinants [1,2,65]. We show an analogous example in meiotic cells where polarity factors localize to the centrosome—telomere cluster cytoplasm in the zygotene bouquet stage. Importantly, before the bouquet stage, the centrosome is at a perinuclear position, but telomeres are not clustered on the NE and Bb components are radially distributed in the cytoplasm. Only when the centrosome associates with the telomeres in the bouquet stage do Bb precursor components begin to aggregate in the telomere cluster cytoplasm. This suggests a mechanistic connection between the centrosome and the clustered telomeres of the bouquet that is also required for the initiation of Bb component recruitment.

In the bouquet stage of various species, telomeres are connected to the centrosome via NE-resident Sun-KASH domain proteins and cytoplasmic microtubules [28,29,31,32,55]. However, the molecular nature of the centrosome—telomere association in the zebrafish meiotic—vegetal center, as well as the functional interactions that recruit Bb components to establish polarity in any vertebrate have been unknown. We analyzed microtubule localization in zebrafish zygotene oocytes and found that the bouquet centrosome—microtubule—telomere cellular organization is conserved. Furthermore, we find that microtubules are simultaneously required for both the telomere clustering and the apposing localization of Bb precursors of the bouquet. In their absence, telomeres and Bb mitochondria are dispersed. Thus, bouquet microtubules provide a mechanistic link between the nuclear events of the bouquet and the cytoplasmic events of Bb formation.

How microtubules localize Bb precursors is still an open question. Microtubules could localize Bb precursors either by movements that stir cytoplasm towards the centrosome or by serving as routes for dynein/kinesin motors that localize Bb precursors as cargo. The (−) end motor dynein is a more likely candidate since localization is towards the (−) end of microtubules at the centrosome. Intriguingly, dynein localizes to telomere-bound Sun/KASH proteins on the NE and is required for chromosome synapsis in *C*. *elegans* zygotene oocytes [31]. More experiments are required to distinguish between these possibilities in zebrafish.

Previous studies in Xenopus found no requirement for microtubules in localizing mRNA to the Bb [66]. However, these experiments were performed at much later stages of oogenesis addressing localization to the mature Bb. Our experiments show that microtubules are required for the initial localization of Bb precursors during its early formation. We also find a microtubule network at pachytene—early diplotene around the centrosome within the nuclear cleft, where Bb precursors are enriched. It will be interesting to address the roles of microtubules in Bb aggregation and cleft formation during these stages.

The tracing of oocyte symmetry breaking to the meiotic—vegetal center in turn raises the question of upstream regulation of the activation and positioning of its components, i.e., the centrosome and the site of clustered telomeres. Germ cells, from insects to humans, develop in germline cysts or nests [44]. While only premeiotic oogonia were described in zebrafish cysts [38], we found that leptotene and zygotene oocytes develop within nests as well, some of which are clearly still connected by cytoplasmic bridges at the zygotene to pachytene transition (Fig 5B–5D).

The synchronized development and polarization of oocytes within the nest provides an environment wherein a higher order organization of the nest could regulate their polarization. The nest could, for example, be organized by intrinsic positioning of the centrosome by preceding oriented cell divisions and/or by paracrine signaling from surrounding follicle cells. We found a novel cytoskeletal feature in the meiotic nest that argues for a higher order nest organization. We find cables of acetylated microtubules in leptotene to zygotene stages and then nonacetylated microtubules at the zygotene to pachytene transition that can extend between two oocytes in the nest, likely reflecting cytoplasmic bridges. This is strikingly similar to the typical events of late cytokinesis, where acetylated midbody microtubules in the cytoplasmic bridge are then deacetylated in order to complete cytokinesis [58–63]. This suggests that the two connected oocytes that we observe constitute daughter cells from the last mitotic division. Remarkably, these microtubule extensions connect the two cells via their centrosome cytoplasm, indicating that the last mitotic division plane may localize the centrosomes and the meiotic—vegetal center, and predispose the axis of the bouquet and oocyte polarization. While no asymmetry is observed in oogonia, polarization may be established in two stages. In this model, the last mitotic division presets the polarization axis, but then polarization is only active at zygotene, when Bb precursors first asymmetrically localize (Fig 7B).

At zygotene stage, the cables of acetylated tubulin associate with centrosomes of the bouquet (Fig 5B and 5C, S6 Fig), where dynamic perinuclear microtubules also reside. The structure of the acetylated cables strikingly resembles that of the axoneme of primary cilia [67]. Similar to primary cilia, these structures in the nest may serve mechanical and/or signaling roles [67]. Such cables may be affixed to the midbody (as discussed above) and serve as anchors or transmit forces important in the dramatic chromosomal movements that generate the zygotene bouquet configuration [31,41].

Forward genetic screens have not identified the oocyte polarity regulators in early meiotic stages in vertebrates [68,69], and the accessibility of these stages to in vivo and quantitative imaging techniques has been challenging. Our dissection of early oocyte polarization in zebrafish now enables a reverse genetic approach, utilizing the advances in CRISPR/Cas9 techniques. Our work redefines the zygotene bouquet as a global cellular organizer at the nexus of oocyte differentiation. The juvenile zebrafish ovary arises as an attractive model to study the intersection of the fundamental biological processes of cell polarity and spatiotemporal tissue organization, encompassing mitosis and meiosis in a key developmental context.

## Materials and Methods

### Fish Lines and Ovary Collections

Ovaries were collected from 6–8 wk postfertilization (wpf) juvenile fish: TU wild type, *buc*^*p43btmb/p43btmb*^ (referred to as *buc*^*-/-*^) [50], *Tg(Ef1α*:*Dclk-GFP*) [6], and *Tg(βAct*:*emtb-3GFP)* [56]. Fish had a standard length (SL) measured according to [70] and were consistently ~12–20 mm. To fix the ovaries for immunostaining, RNA-FISH, and DNA-FISH, fish were cut along the ventral midline and the lateral body wall was removed. The head and tail were removed and the trunk pieces, with the exposed abdomen containing the ovaries, were fixed in 4% PFA at 4°C overnight with nutation. Trunks were then washed in PBS, and ovaries were finely dissected in cold PBS. Ovaries were washed in PBS and then either stored in PBS at 4°C in the dark, or dehydrated and stored in 100% MeOH at −20°C in the dark. For microtubule staining, fixation was carried out using a microtubule stabilizing buffer (MSB), including 3.7% formaldehyde, 0.25% glutaraldehyde, and 0.5 μM Taxol (MSB-fix; [71]). Post-fix washes and dissections were done in MSB without fix and taxol, and ovaries were stored in MeOH at −20°C in the dark.

### Fluorescence Immunohistochemistry (IHC), RNA-FISH, and DNA-Telo-FISH

Ovaries were washed 2 times for 5 min (2 x 5 min) in PBT (0.3% Triton X-100 in 1 x PBS; if stored in MeOH, ovaries were gradually rehydrated first), then washed 2 x 20 min, 1 x 40 min, 1 x 20 min in PBT. Ovaries were blocked for 1.5–2 h in blocking solution (10% FBS in PBT) at room temperature and then incubated with primary antibodies in blocking solution at 4°C overnight. Ovaries were washed 4 x 20 min in PBT and incubated with secondary antibodies in fresh blocking solution for 1.75 hr and were light protected from this step onward. Ovaries were washed 4 x 20 min in PBT and then incubated in PBT containing DAPI (1:1,000, Molecular Probes), with or without DiOC6 (1:5,000, Molecular Probes) for 50 min and washed 2 x 5 min in PBT and 2 x 5 min in PBS. All steps were carried out with nutation. Ovaries were transferred into Vectashield (with DAPI, Vector labs). Ovaries were finally mounted between two #1.5 coverslips using a 120 μm spacer (Molecular Probes).

Primary antibodies used were mAb414 (1:1,000, Abcam), LamB1 (1:400, Abcam), γTubulin (1:400, Sigma-Aldrich), Buc (1:500) [34], LamA/C (ready for use, Progen Biotech), GFP (1:400; Molecular Probes), Vasa (1:5,000) [72], Zili (1:100) [73], Ziwi (1:250) [74], GasZ (1:100) [45], Acetylated tubulin (1:200; Sigma-Aldrich), β-Catenin (1:1,000; Sigma-Aldrich). Secondary antibodies used were anti-rabbit IgG, or anti-mouse IgG1, or anti-mouse IgG2b, Alexa 488, and Alexa 594 (all 1:500, Molecular Probes).

RNA-FISH was performed using the DNA-HCR-FISH technique (Molecular Instruments) [75], following the company protocol, except for the hybridization temperature that was optimized for 50°C.

DNA-FISH for telomeric repeats (Telo-FISH) was performed using the PNA technique (PNA-Bio) following the company protocol. Hybridization buffer was 70% Formamide, 1 mM Tris pH 7.2, 8.5% MgCl2 buffer (25 mM magnesium chloride, 9 mM citric acid, 82 mM sodium hydrogen phosphate, pH7), 1x Blocking reagent in Maleic acid buffer (100 mM Maleic acid, pH7.5), 0.1% Tween20, 88 nM probe (*5’-CCCTAACCCTAACCCTAA-3’*, Cy3- conjugated).

For a combination of IHC with RNA-FISH or Telo-FISH, IHC was performed first. At the end of the IHC procedure, ovaries were washed an extra time for 30 min in PBT and fixed quickly in 4% PFA for 15–20 min at room temperature. Ovaries were washed 3 x 5 min in PBS. If FISH procedures did not start immediately, ovaries were stored overnight in PBS at 4°C. During IHC that preceded RNA-FISH, all blocking solutions also contained RNAasin (1:100, Sigma-Aldreich), to protect against potential RNAases in the blocking serum. All transitions between SSC buffers and PBS were done gradually to protect tissue morphology. After staining was complete, DAPI (+/- DiOC6) staining and mounting was performed as described above.

### Confocal Microscopy, Image Acquisition, and Processing

Images were acquired on a Zeiss LSM 710 confocal microscope using a 40X lens. The acquisition setting was set between samples and experiments to: XY resolution = 1,104 x 1,104 pixels, 12-bit, 2x sampling averaging, pixel dwell time = 0.59 sec, zoom = 0.8X, pinhole adjusted to 1.1 μm of Z thickness, increments between images in stacks were 0.53 μm, laser power and gain were set in an antibody-dependent manner to 7%–11% and 400–650, respectively, and below saturation condition. Acquired images were not manipulated in a nonlinear manner, and only contrast and brightness were adjusted. All figures were made using Adobe Photoshop CC 2014.

### Image Quantification

Measurements were performed using a custom MATLAB (Version 8.2.0.701 R2013b 64-bit, MathWorks) script.

#### Cleft analysis

We observed that in pachytene-early diplotene oocytes (17–25 μm) the centrosome localizes roughly to the center of the cleft (Fig 2B) and roughly to the center of the Bb precursor aggregate within the cleft (S5A Fig). Based on these observations, we developed the code below, which utilizes the centrosome as a marker for cleft cytoplasm and measures intensities in “in-cleft cytoplasm” (centrosome-adjacent cytoplasm) versus “noncleft cytoplasm” (centrosome-nonadjacent cytoplasm).

Pachytene and early diplotene oocytes were manually staged according to the criteria in Fig 2. A region of interest (ROI) was hand-drawn on each slice of the three-dimensional (3-D) image stack to outline each oocyte. Within this ROI, subregions of the oocyte were identified by reference stain intensity. First, nuclear regions were defined as areas of the cell where DAPI was above a user-specified threshold, and gamma tubulin background staining (which is absent from the nucleus) was below a user-specified threshold (S5B Fig, left column). All nuclear regions were subtracted from the ROI to specifically yield the cytoplasmic region (S5B Fig, center column).

Next, we identified the centrosome and split the cytoplasm into a region close to the centrosome (“centrosome-adjacent”) and a region far from the centrosome (“centrosome-nonadjacent”). We defined a centrosome to be a three-dimensional (3-D) group of at least eight connected pixels with gamma tubulin staining above a minimum threshold. The center point of the 3-D pixel group was identified using the regional maxima of a Euclidean distance transform. To select the centrosome-adjacent cytoplasm, a sphere (radius = 4.8 μm) was drawn around the center of the centrosome. We called the cytoplasm within this sphere centrosome-adjacent (S5B Fig, right column, Fig 1B), and it represents “in-cleft cytoplasm.” To select centrosome-nonadjacent cytoplasm, a larger sphere (radius = 6 μm) was drawn around the center point of the centrosome. We called the cytoplasm outside of this sphere centrosome-nonadjacent (S5B Fig, right column; Fig 1B) and it represents “noncleft cytoplasm". The sphere radii were chosen by measuring the approximate sizes of clefts in most oocytes and assigning a radius that most often captured the cytoplasm within the cleft.

The average pixel intensity of the experimental stain (*dazl* mRNA, Buc, or DiOC_6_) was measured in each region (nuclear, centrosome-adjacent cytoplasm, and centrosome-nonadjacent cytoplasm). To normalize pixel intensity across different 3-D image stacks, the average pixel intensity within the nuclear region was subtracted from the cytoplasmic intensities.

#### Zygotene nest analysis

We found that the centrosome localizes to the telomere cluster cytoplasm in zygotene oocytes (Fig 2B) and can thus serve as a marker for this region. We also observed that, similar to premeiotic oogonia oocytes, zygotene oocytes reside in nests, where they are tightly clustered, with follicle cells collectively surrounding the clustered oocytes in each nest (Fig 2C and 2D, S7 Video). Because nests of zygotene oocytes are packed tightly, analysis of individual zygotene oocytes was challenging and potentially inaccurate. We instead developed the code described below to measure intensities in centrosome-adjacent cytoplasm (“telomere cluster cytoplasm”) versus the centrosome-nonadjacent cytoplasm (the remaining cytoplasm away from the telomere cluster) in entire zygotene nests, analyzing many cells simultaneously.

Nests of zygotene oocytes were identified manually, according to the criteria in Fig 2 and S3 Fig. A region of interest (ROI) was hand-drawn around the zygotene oocytes of interest on each slice of the three-dimensional (3-D) image stack. These ROI’s may contain one or multiple zygotene nests. The close packing of these nests made it difficult to unambiguously distinguish between one nest and the next. Individual oocytes were not separated in this analysis, and the cytoplasm of the cells in the entire nest or groups of nests was measured in bulk. To identify specific subregions of the nest, first nuclei were defined by the presence of DAPI staining above a user-specified threshold and the absence of cytoplasmic background gamma tubulin staining defined by a user-specified threshold (S5C Fig, left column). All nuclear regions were subtracted from the ROI to specifically yield the cytoplasmic region (S5C Fig, center column).

Next, we identified the centrosome and split the cytoplasm into regions close to centrosomes (“centrosome-adjacent”) and regions far from centrosomes (“centrosome-nonadjacent”). We defined a centrosome to be a three-dimensional (3-D) group of at least eight connected pixels with gamma tubulin staining above a minimum threshold. The center point of the 3-D pixel group was identified using the regional maxima of a Euclidean distance transform. To select the centrosome-adjacent cytoplasm, a sphere was drawn around the center of all centrosomes within the ROI (radius = 1.2 μm). The approximate distance between the centrosome and the nucleus in most cells determined the radius of the sphere. Cytoplasm within these spheres was defined as centrosome-adjacent (S5C Fig, right column). To define the centrosome-nonadjacent cytoplasm, a larger sphere was drawn around the center of all centrosomes in the image (radius = 2.4 μm), and any cytoplasm not encompassed by these spheres was called centrosome-nonadjacent (S5C Fig, right column).

The average pixel intensity of the experimental stain (Buc or DiOC6, or Dclk-GFP) was measured in each region (nuclear, centrosome-adjacent cytoplasm and centrosome-nonadjacent cytoplasm). To normalize pixel intensity across different 3-D image stacks, the average pixel intensity within the nuclear region was subtracted from the cytoplasmic intensity.

### GasZ and mAb414 Colocalization

A pixel-wise quantitative test for colocalization of GasZ with mAb414 signals was performed using the Coloc 2 plug-in on Fiji. We calculated a point spread function (PF) of ~2.5–3 pixels on average for each channel and image, based on PSF = [0.8 x Exitation wave length (nm) / objective’s NA] / pixel size (nm). Min and max thresholds were automatically set by the plug-in for each image. Pearson correlation coefficient (R) was calculated to be ~0.5. R values represent: −1 < R < 0, anticorrelation; R = 0, no correlation; 0 < R < 1, correlation. The plug-in then scrambles the pixels to generate a random image and test for the probability to yield the same Pearson coefficient from a random image. Scrambling was set to 100 iterations, generating 100 different random images from the tested image’s pixels. The probability of receiving Pearson coefficient of R = ~0.5 was *p* = 1. The plug-in also yielded Manders coefficient, which represents the colocalized cohort of each signal (here, for each GasZ and mAb414 was ~75%).

### Images of Live Whole Ovaries

Ovaries were dissected from juvenile fish (6–8 wpf, SL ~12–20 mm) into fresh warm HL-15 media (Hanks solution containing 60% L-15 (no phenol red), Sigma-Aldrich, and 1:100 Glutamax, Invitrogen, warmed to 28°C). Ovaries were then embedded in 0.5% low-melt agarose in HL-15 on a glass-bottom dish, and covered with warm HL-15. After the agarose polymerized, ovaries were incubated in HL-15 containing Hoechst (6.66 μM, Molecular Probes), DiOC6 (1:5,000), and/or, Mitotracker (500 nM, Molecular Probes), for 2 hr at 28°C. Still images of live ovaries were acquired using the Zeiss LSM 710 confocal microscope and a 40X lens with the settings described above.

### Nocodazole Treatments

Ovaries were dissected from WT or *Tg(βAct*:*emtb-3GFP)* juvenile fish (6–8 wpf, SL ~12–20 mm) into fresh warm HL-15 media. Then HL-15 was replaced with HL-15 containing either 100 μM nocodazole (EMD Millipore) or an equivalent volume of DMSO. The two ovaries from each fish were split between the nocodazole and DMSO groups as an internal control. Ovaries were incubated for 80 min at 28°C in the dark. Ovaries were then washed twice with plain HL-15, and fixed either with 4%PFA/PBS, or with MSB-fix as described above. Ovaries were stored and stained as described above. To determine these conditions, we performed a time-lapse live imaging of *Tg(βAct*:*emtb-3GFP)* ovaries monitoring microtubules. We tested several nocodazol and DMSO concentrations and determined the incubation time for our experiments by the earliest time point in the movies that showed efficient microtubule depolymerization. Ovaries were still healthy and viable for at least 8 h after the 80-min time point. DMSO-treated ovaries always showed normal intact microtubules.

### Electron Microscopy

Ovaries were dissected from juvenile fish (6–8 wpf, SL ~12–20 mm) and fixed as described above in 2% PFA and 2.5% gluteraldehyde. To maximally preserve the tissue morphology, samples were prepared using a high pressure freezing technique [76]. Ovaries were vitrified under high pressure liquid nitrogen in an Abra HPM010. Ovaries were freeze substituted in 2% OsO4, 0.1% U acetate, in 100% acetone at −90°C for 72 hr. After slowly warming to room temperature, the ovaries were infiltrated and embedded in EMBed-812 (Electron Microscopy Sciences). Thin sections were taken, counterstained with uranyl acetate and lead citrate, and examined with a JEOL 1010 electron microscope fitted with a Hamamatsu digital camera, and using AMT Advantage image capture software.

For EM images, the “levels” and “brightness/contrast” functions on Adobe Photoshop, were mildly adjusted to improve the image tones and make the image generally crisper. These adjustments did not affect the biological properties of the imaged cellular features.

### QRT-PCR

WT or *buc*^*-/-*^ ovaries (SL = 17–22 mm) were dissected and immediately transferred into Trizol (Life Technologies; four ovaries were pooled of each genotype) and snapfrozen and stored at −80°C. Total RNA was extracted using Trizol and chloroform extractions, followed by DNAaseI reaction. RT on WT, *buc*^*-/-*^ and (−RT) control (from a WT RNA sample) sample was performed using random hexamer primers and the Superscript kit (Invitrogen), followed by RNAaseH reaction according to the kit instructions. QPCR was performed using SYBR Green Jumpstart Taq Ready Mix (Sigma-Aldrich), in Step One Plus PCR machine (Applied Biosystems). Reactions on WT, *buc*^*-/-*^ and (−RT), and no-template controls were performed in duplicates, and each reaction was repeated twice. *dazl* levels, normalized to *βActin*, were compared using the ΔΔC_T_ method.

### Statistical Analysis

All statistical analysis and data plotting was performed using the GraphPad Prism 6 software. Data sets were tested with two-tailed unpaired *t* test. *p*-values were: *<0.05, **<0.01, ****<0.0001, ns = not significant (>0.05).

## Supporting Information

S1 Data Supporting information for Figs 1B, 3A, 3B, 3D and 3E, 4C and 6F and S3B and S4B Figs. The numerical values of the data presented in these figures are provided. Data for each figure is found as an individual spreadsheet in this Excel file.
(XLSX)

S1 Fig A montage of the full stack of images of the oocyte shown in Fig 1B. Staining: *dazl* (green), or Buc, or DiOC6, DAPI (blue). Measurements lines: oocyte outline (white), noncleft cytoplasm (red), cleft cytoplasm (green). See cleft analysis in S5 Fig.
(TIF)

S2 Fig The nuclear cleft is a feature of the early oocyte. **(A)** The nuclear cleft, as visualized with LamB1, is enriched with the Bb precursor *dazl* RNA. Diplotene onset (top), pachytene (bottom). S2 Video shows the full stack of images of these oocytes, and S3 Video shows their 3-D view. **(B)** TEM images of representative ~20 μm (μ), ~20–25 μm and ~30 μm oocytes showing the typical nuclear cleft for these stages. The colors of framed regions in lower magnification images match the frame colors of the corresponding higher magnification images. Blue and green frames are higher magnification views of in-cleft cytoplasm. Red and yellow frames are higher magnification views of noncleft cytoplasm. The NE (yellow arrowheads) is concave forming the cleft, which is enriched with mitochondria (yellow arrows are examples of mitochondria) and electron-dense material presumably detecting mRNPs (*). Non-cleft cytoplasm contains presumptive mRNPs but is not enriched with mitochondria, and its adjacent NE is not concave. Cyto, cytoplasm; Nuc, nucleus. Note the nuclear peninsulas in the more pronounced cleft of the ~20 μm oocyte. In the ~20–25 μm oocyte, the cleft appears to be perpendicular to the image plane, with a view into the cleft. Note the cytoplasm (red *) engulfed by the nuclear protrusions and the presumptive mRNP clusters (green arrowheads) all around the NE inside the cleft. On the confocal microscope, as shown here in TEM, the nuclear cleft morphology of the 30 μm oocyte is consistently milder. Scale bars are indicated. **(C)** LamA/C is specifically detected in postcleft oocytes where the nucleus resumes a spherical shape (red arrowheads). Cleft stages are marked with white arrowheads. DiOC6 detects the concave NE and the cleft-enriched mitochondria. S4 Video shows the complete confocal stack. LamB1 is expressed in cleft (panel A; Fig 1C) and postcleft stages.
(TIF)

S3 Fig Precise criteria for staging early meiotic zebrafish oocytes. **(A)** Top: Images of the entire oocytes in Fig 2A, also showing DiOC6, which labels the cytoplasm and makes evident the size of the oocyte. Bottom: Nuclear zoom-in views of the same oocytes. DAPI (greyscale) show chromosome morphology. Range of sizes for specific stages is plotted in **(B)**. Oocytes from 3–4 ovaries per stage were measured. Data in S1 Data. **(C)** Additional nuclear morphological criteria for each stage, including nucleolus number and positions, as well as DNA condensation, state characteristic of these early stages. These criteria expand upon the previously described characteristics of early oocytes in the zebrafish [38–40].
(TIF)

S4 Fig Supporting information for Fig 3. **(A)** Quantification of mitochondrial enrichment in zygotene bouquet TEM image overlapping high magnification images of the entire cytoplasm 360° around the nucleus of the cell in the top panel are shown in the lower panels. Image numbers (bottom) correspond to the numbered regions (top). SC-NE contact points (yellow arrowheads) mark the presumptive telomere cluster. Mitochondria (examples are marked by yellow arrows) were counted in regions adjacent to SC-NE contact points versus regions that are not. In this example, frames 4–6 span the presumptive telomere cluster. Mitochondria are mostly found in the cytoplasm apposing this region versus that of frames 1–3 and 7–8. Pooled data are plotted in Fig 4A (*n* = 7 oocytes). Zygotene oocytes were identified by their size (measurements are shown in A), the detection of SC, and the typical peripheral nucleolus. Nuc, nucleus; Cyto, cytoplasm; nucl, nucleolus. Green arrowhead, NE; red arrowhead, cell membrane. Red arrowheads in the entire cell (top) indicate to the same regions in the corresponding smaller higher power images. **(B)** Zygotene nest analysis for DCLK-GFP, as shown in Fig 3B for DiOC6 and Buc. DCLK appears randomly radially distributed and shows no enrichment in the centrosome cytoplasm of either oogonia or zygotene oocytes (*n* = 5 ovaries ‒ Zygotene, *n* = 26 oocytes in 4 nests, Oogonia, *n* = 6 oocytes in 2 nests). Data in S1 Data. **(C)** mAb414 detects the NE and colocalizes with perinuclear granules in zebrafish oocytes. The mAb414 perinuclear spherules signal (distinct from the fine line of the NE) colocalizes with the piRNA-specific proteins Vasa (*n* = 8 ovaries), GasZ (*n* = 7 ovaries), Zili, (*n* = 4 ovaries), and Ziwi (*n* = 4 ovaries), here shown in oogonia. GasZ oocyte shown is the same cell shown in Fig 3C, but also showing the different channels separately. **(D)** GasZ is a Bb resident protein during mid-to-late diplotene stages. GasZ colocalizes with *dazl* mRNA (costained with DAPI, blue) in the mature Bb (arrowheads; *n* = 8 ovaries).
(TIF)

S5 Fig Supporting information for cleft and nest analyses. **(A)** Bb precursor components in the cleft are aggregated around the centrosome. The centrosome localizes to the nuclear cleft throughout pachytene (cleft formation) to early diplotene (≤25 μm). Bb precursors aggregate in the cleft surrounding the centrosome (*dazl*, *n* = 6 ovaries; Buc, *n* = 10 ovaries; DiOC6, Fig 2B). **(B)** Identification of oocyte subregions for the cleft analysis using a MATLAB code. Representative images of pachytene and early diplotene (17–25 μm) oocytes quantified with cleft analysis. Each panel shows four adjacent single z-slices. Identified nuclei (left column) were subtracted to reveal the cytoplasm region only (center column). In the identified overall cytoplasm, subregions of centrosome-adjacent and centrosome-nonadjacent cytoplasm were identified (right column). Staining: experimental stain (green), γTub (red), DAPI (blue). Outlined regions: region of interest (ROI) (white), all nuclei (blue), all cytoplasm (yellow), centrosome-adjacent cytoplasm (green), centrosome-nonadjacent cytoplasm (red). Scale bars are 10μm. **(C)** Identification of oocyte subregions for the nest analysis using a MATLAB code. Representative images of nests of zygotene oocytes quantified with zygotene nest analysis. Each panel shows four adjacent single z-slices. Identified nuclei (left column), cytoplasm region only (center column), and subregions of centrosome-adjacent and centrosome-nonadjacent cytoplasm (right column) are shown as in B. Staining, outlines, and scale bar as in B.
(TIF)

S6 Fig Supporting information for Fig 3. Telo-FISH staining (red) confirms the stage specificity of the acetylated tubulin cables (green) in the nest (costained with DAPI, blue). Premeiotic oogonia with intranuclear scattered telomeres (left) show no acetylated tubulin cables. Leptotene oocytes with telomeres loaded radially on the NE show some cables (center). Zygotene oocytes with tightly clustered telomeres on the NE show more elaborated cables associated with them. *n* = 6 ovaries. Scale bar: 10 μm.
(TIF)

S1 Video A 3-D view of a 25 μm early diplotene oocyte nucleus, showing the nuclear cleft (labeled with mAb414; fine line, NE; spherules, perinuclear granules). The cleft is marked with orange arrows and was enriched with DiOC6 (the DiOC6 channel was omitted for clarity).
(AVI)

S2 Video The full z-stack of images of the projection in S2A Fig. (AVI)

S3 Video A 3-D view of the stack in S2A Video, showing the Bb precursor aggregate (marked with *dazl*, green) intimately within the nuclear cleft (LamB1, red) in the right panel. In the left panel the green *dazl* channel was omitted, showing the exposed nuclear cleft (green arrows).
(MP4)

S4 Video The full z-stack of images of the projection in S2C Fig. LamA/C (red) is only detected in nuclei of postcleft oocytes (red arrows), and not in cleft stages oocytes (white arrows). DiOC6 (green) detects the NE, as well as organelle membranes, including the enriched mitochondria in the cleft.
(AVI)

S5 Video A 3-D view of the bouquet configuration in an early zygotene oocyte. Note the tight clustering of telomeres (red; DAPI, cyan) forming a cap in the nuclear periphery.
(AVI)

S6 Video A 3-D view of the bouquet configuration in a late zygotene oocyte. Note that telomeres (red; DAPI, cyan) are still positioned to one pole of the nucleus but are more scattered than in early zygotene (S5 Video).
(AVI)

S7 Video A 3-D view of a nest of zygotene oocytes. Zygotene oocytes (with clustered telomeres, red) are located together in what is termed a nest, with no other cells between them. Nuclei of follicle cells (more amorphous; DAPI, cyan) are only seen in the periphery of the nest. Note that the cytoplasm is not labeled.
(AVI)

S8 Video A 3-D view of the acetylated tubulin cables and centrosomes in a nest of zygotene oocytes. The complete 3-D view of the nest shown in Fig 3C.
(AVI)

S9 Video A 3-D view of the acetylated tubulin cables and ytoplasmic membranes in a nest of zygotene oocytes. The complete 3-D view of the nest shown in Fig 3D.
(AVI)

## Abbreviations

AV: animal—vegetal
Bb: Balbiani body
Buc: Bucky ball
DMSO: dimethyl sulfoxide
EM: electron micrograph
IDP: intrinsically disordered protein
mRNP: mRNA protein granule
MTOC: microtubule organizing center
NE: nuclear envelope
QRT-PCR: quantitative real-time polymerase chain reaction
ROI: region of interest
SC: synapsed chromosome
SD: standard deviation
SEM: standard error of the mean
TEM: transmission electron microscopy
Wt: wild-type
