# Supplementary figures and images for: Oocyte Polarization Is Coupled to the Chromosomal Bouquet, a Conserved Polarized Nuclear Configuration in Meiosis
Source: PLoS Biol. 2016 Jan 7;14(1):e1002335. doi: 10.1371/journal.pbio.1002335 (PMC4704784; doi:10.1371/journal.pbio.1002335)

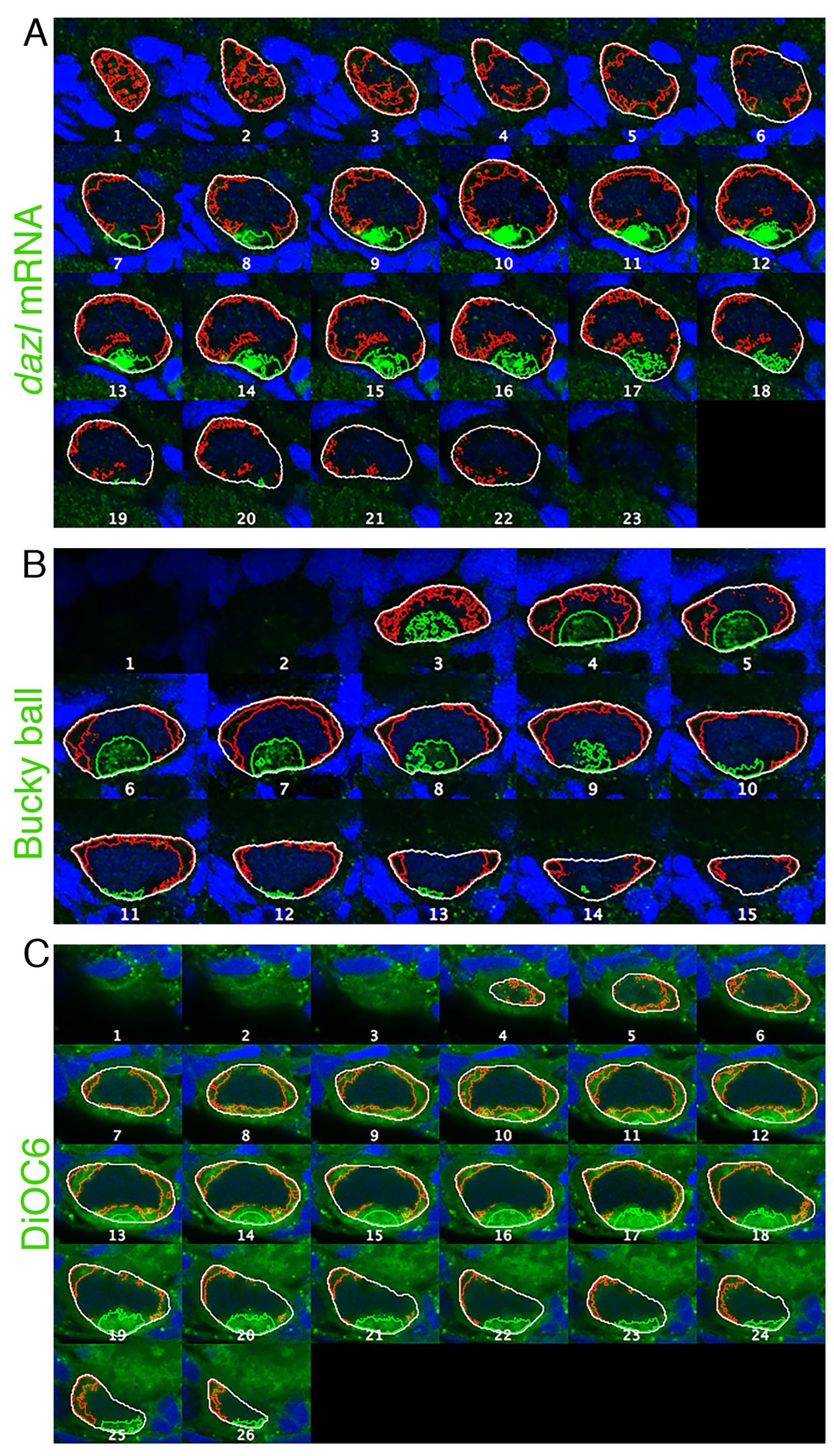

Supplement: S1 Fig — Staining: dazl (green), or Buc, or DiOC6, DAPI (blue). Measurements lines: oocyte outline (white), noncleft cytoplasm (red), cleft cytoplasm (green). See cleft analysis in S5 Fig. (TIF) [file pbio.1002335.s002.tif]

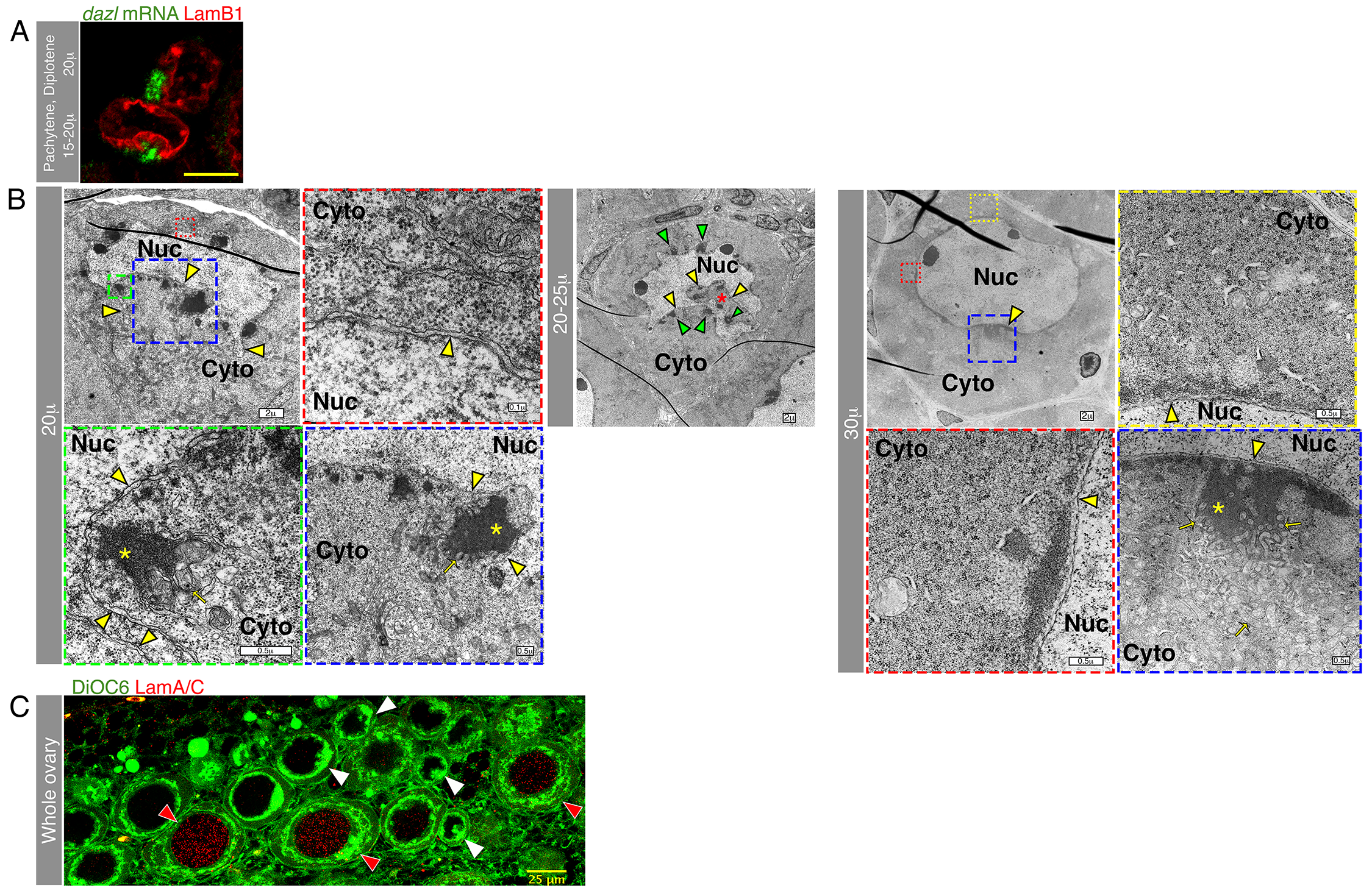

Supplement: S2 Fig — (A) The nuclear cleft, as visualized with LamB1, is enriched with the Bb precursor dazl RNA. Diplotene onset (top), pachytene (bottom). S2 Video shows the full stack of images of these oocytes, and S3 Video shows their 3-D view. (B) TEM images of representative ~20 μm (μ), ~20–25 μm and ~30 μm oocytes showing the typical nuclear cleft for these stages. The colors of framed regions in lower magnification images match the frame colors of the corresponding higher magnification images. Blue and green frames are higher magnification views of in-cleft cytoplasm. Red and yellow frames are higher magnification views of noncleft cytoplasm. The NE (yellow arrowheads) is concave forming the cleft, which is enriched with mitochondria (yellow arrows are examples of mitochondria) and electron-dense material presumably detecting mRNPs (*). Non-cleft cytoplasm contains presumptive mRNPs but is not enriched with mitochondria, and its adjacent NE is not concave. Cyto, cytoplasm; Nuc, nucleus. Note the nuclear peninsulas in the more pronounced cleft of the ~20 μm oocyte. In the ~20–25 μm oocyte, the cleft appears to be perpendicular to the image plane, with a view into the cleft. Note the cytoplasm (red *) engulfed by the nuclear protrusions and the presumptive mRNP clusters (green arrowheads) all around the NE inside the cleft. On the confocal microscope, as shown here in TEM, the nuclear cleft morphology of the 30 μm oocyte is consistently milder. Scale bars are indicated. (C) LamA/C is specifically detected in postcleft oocytes where the nucleus resumes a spherical shape (red arrowheads). Cleft stages are marked with white arrowheads. DiOC6 detects the concave NE and the cleft-enriched mitochondria. S4 Video shows the complete confocal stack. LamB1 is expressed in cleft (panel A; Fig 1C) and postcleft stages. (TIF) [file pbio.1002335.s003.tif]

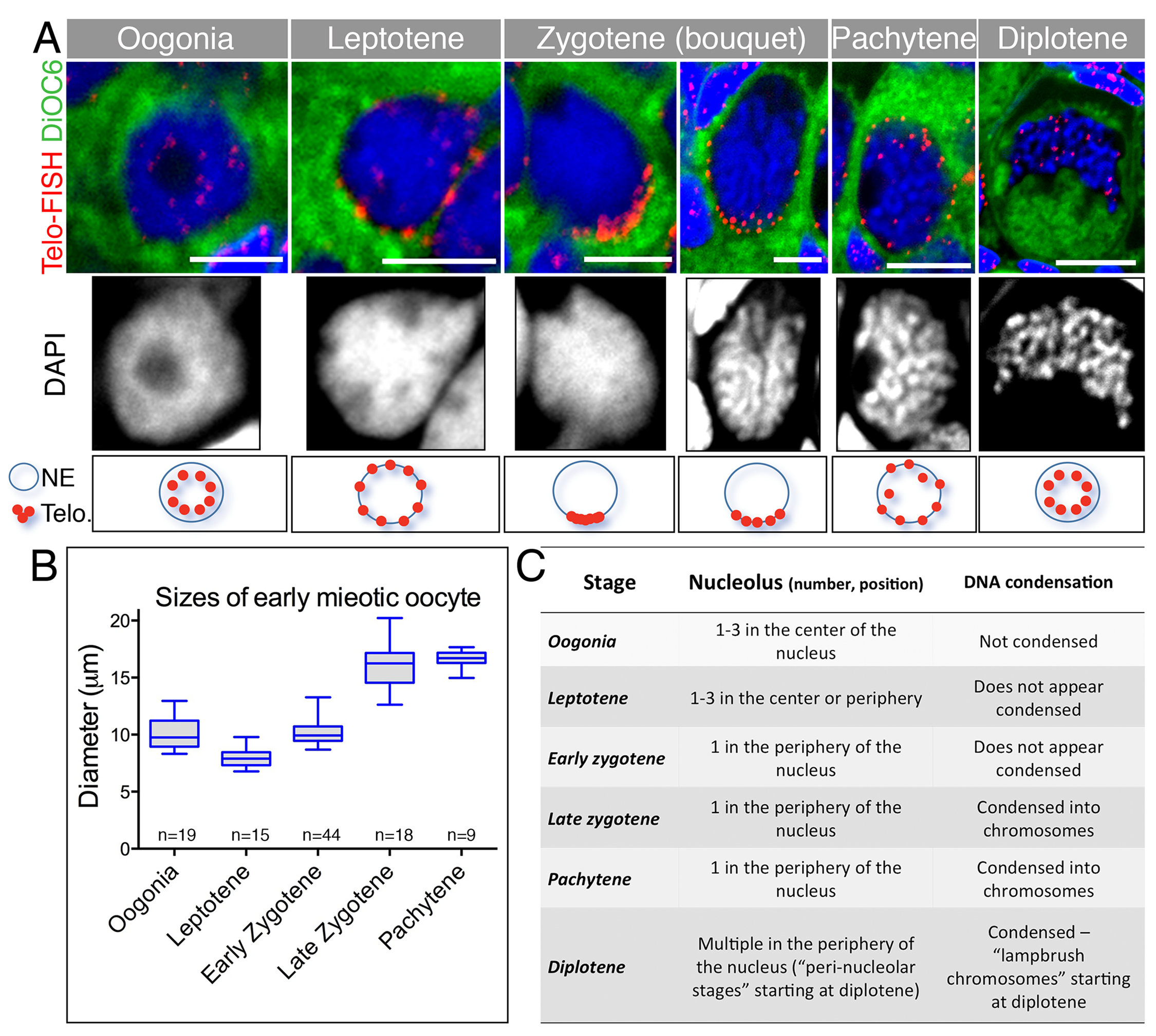

Supplement: S3 Fig — (A) Top: Images of the entire oocytes in Fig 2A, also showing DiOC6, which labels the cytoplasm and makes evident the size of the oocyte. Bottom: Nuclear zoom-in views of the same oocytes. DAPI (greyscale) show chromosome morphology. Range of sizes for specific stages is plotted in (B). Oocytes from 3–4 ovaries per stage were measured. Data in S1 Data. (C) Additional nuclear morphological criteria for each stage, including nucleolus number and positions, as well as DNA condensation, state characteristic of these early stages. These criteria expand upon the previously described characteristics of early oocytes in the zebrafish [38–40]. (TIF) [file pbio.1002335.s004.tif]

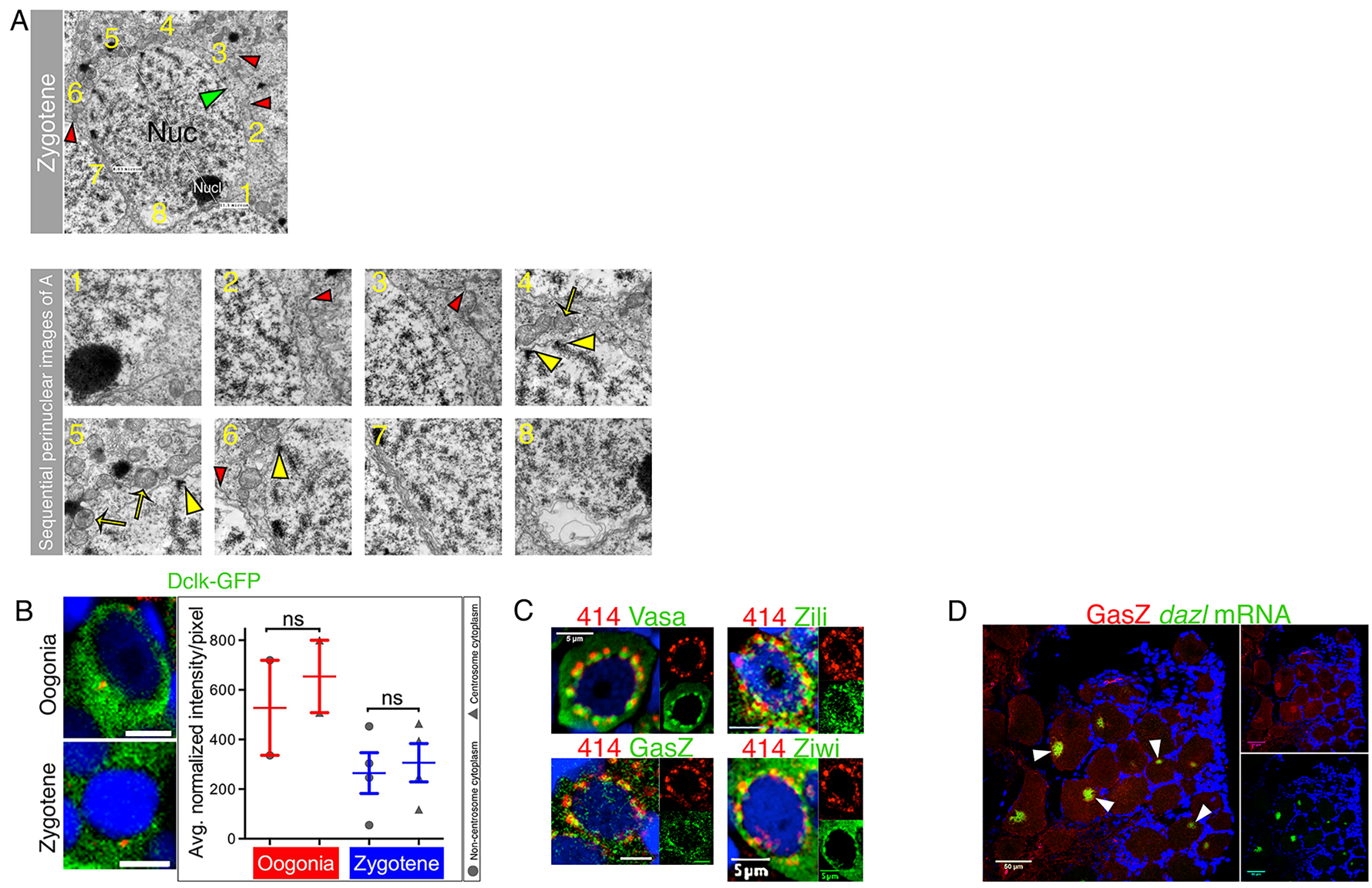

Supplement: S4 Fig — (A) Quantification of mitochondrial enrichment in zygotene bouquet TEM image overlapping high magnification images of the entire cytoplasm 360° around the nucleus of the cell in the top panel are shown in the lower panels. Image numbers (bottom) correspond to the numbered regions (top). SC-NE contact points (yellow arrowheads) mark the presumptive telomere cluster. Mitochondria (examples are marked by yellow arrows) were counted in regions adjacent to SC-NE contact points versus regions that are not. In this example, frames 4–6 span the presumptive telomere cluster. Mitochondria are mostly found in the cytoplasm apposing this region versus that of frames 1–3 and 7–8. Pooled data are plotted in Fig 4A (n = 7 oocytes). Zygotene oocytes were identified by their size (measurements are shown in A), the detection of SC, and the typical peripheral nucleolus. Nuc, nucleus; Cyto, cytoplasm; nucl, nucleolus. Green arrowhead, NE; red arrowhead, cell membrane. Red arrowheads in the entire cell (top) indicate to the same regions in the corresponding smaller higher power images. (B) Zygotene nest analysis for DCLK-GFP, as shown in Fig 3B for DiOC6 and Buc. DCLK appears randomly radially distributed and shows no enrichment in the centrosome cytoplasm of either oogonia or zygotene oocytes (n = 5 ovaries ‒ Zygotene, n = 26 oocytes in 4 nests, Oogonia, n = 6 oocytes in 2 nests). Data in S1 Data. (C) mAb414 detects the NE and colocalizes with perinuclear granules in zebrafish oocytes. The mAb414 perinuclear spherules signal (distinct from the fine line of the NE) colocalizes with the piRNA-specific proteins Vasa (n = 8 ovaries), GasZ (n = 7 ovaries), Zili, (n = 4 ovaries), and Ziwi (n = 4 ovaries), here shown in oogonia. GasZ oocyte shown is the same cell shown in Fig 3C, but also showing the different channels separately. (D) GasZ is a Bb resident protein during mid-to-late diplotene stages. GasZ colocalizes with dazl mRNA (costained with DAPI, blue) in the mature Bb (arrowheads; n = [file pbio.1002335.s005.tif]

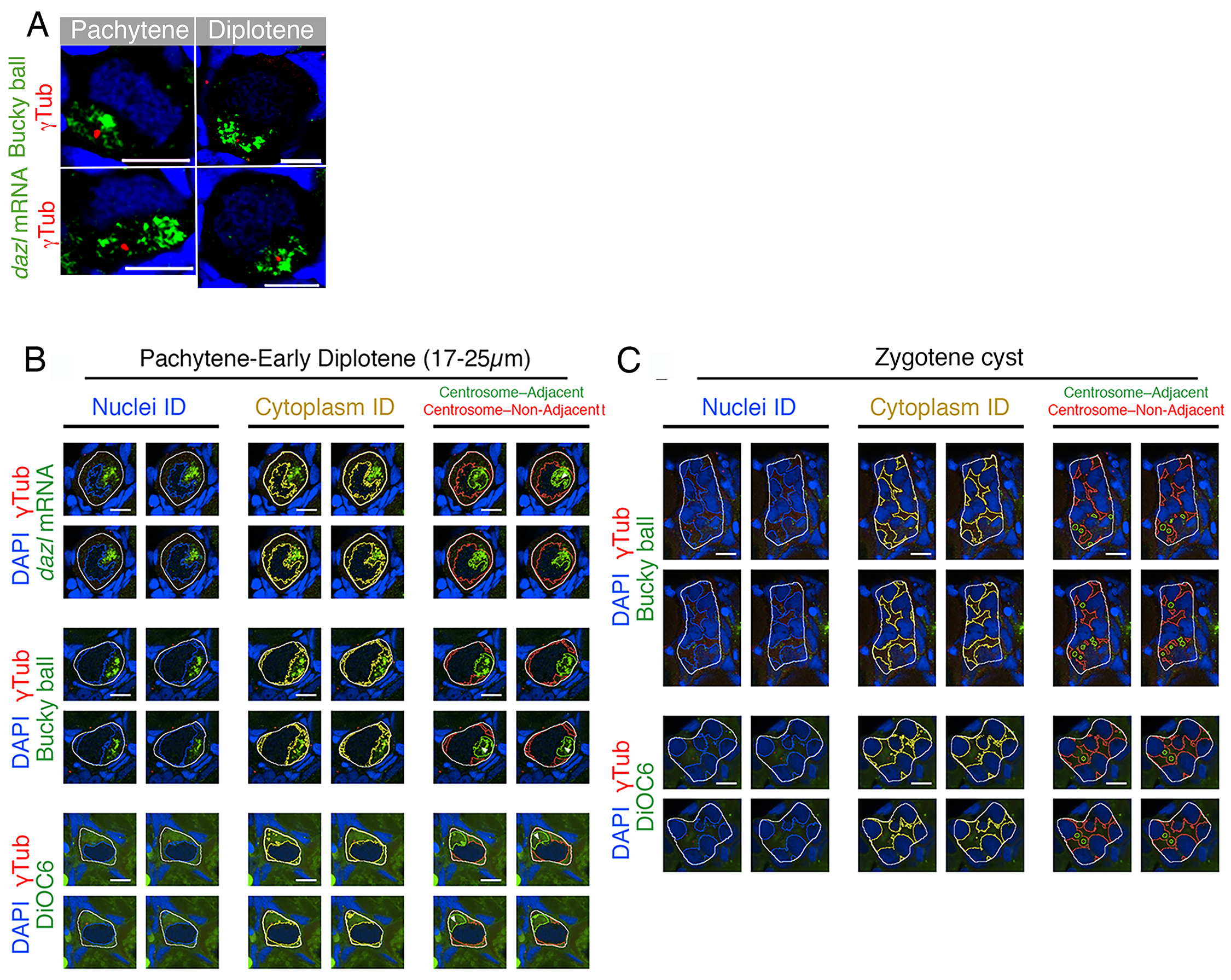

Supplement: S5 Fig — (A) Bb precursor components in the cleft are aggregated around the centrosome. The centrosome localizes to the nuclear cleft throughout pachytene (cleft formation) to early diplotene (≤25 μm). Bb precursors aggregate in the cleft surrounding the centrosome (dazl, n = 6 ovaries; Buc, n = 10 ovaries; DiOC6, Fig 2B). (B) Identification of oocyte subregions for the cleft analysis using a MATLAB code. Representative images of pachytene and early diplotene (17–25 μm) oocytes quantified with cleft analysis. Each panel shows four adjacent single z-slices. Identified nuclei (left column) were subtracted to reveal the cytoplasm region only (center column). In the identified overall cytoplasm, subregions of centrosome-adjacent and centrosome-nonadjacent cytoplasm were identified (right column). Staining: experimental stain (green), γTub (red), DAPI (blue). Outlined regions: region of interest (ROI) (white), all nuclei (blue), all cytoplasm (yellow), centrosome-adjacent cytoplasm (green), centrosome-nonadjacent cytoplasm (red). Scale bars are 10μm. (C) Identification of oocyte subregions for the nest analysis using a MATLAB code. Representative images of nests of zygotene oocytes quantified with zygotene nest analysis. Each panel shows four adjacent single z-slices. Identified nuclei (left column), cytoplasm region only (center column), and subregions of centrosome-adjacent and centrosome-nonadjacent cytoplasm (right column) are shown as in B. Staining, outlines, and scale bar as in B. (TIF) [file pbio.1002335.s006.tif]

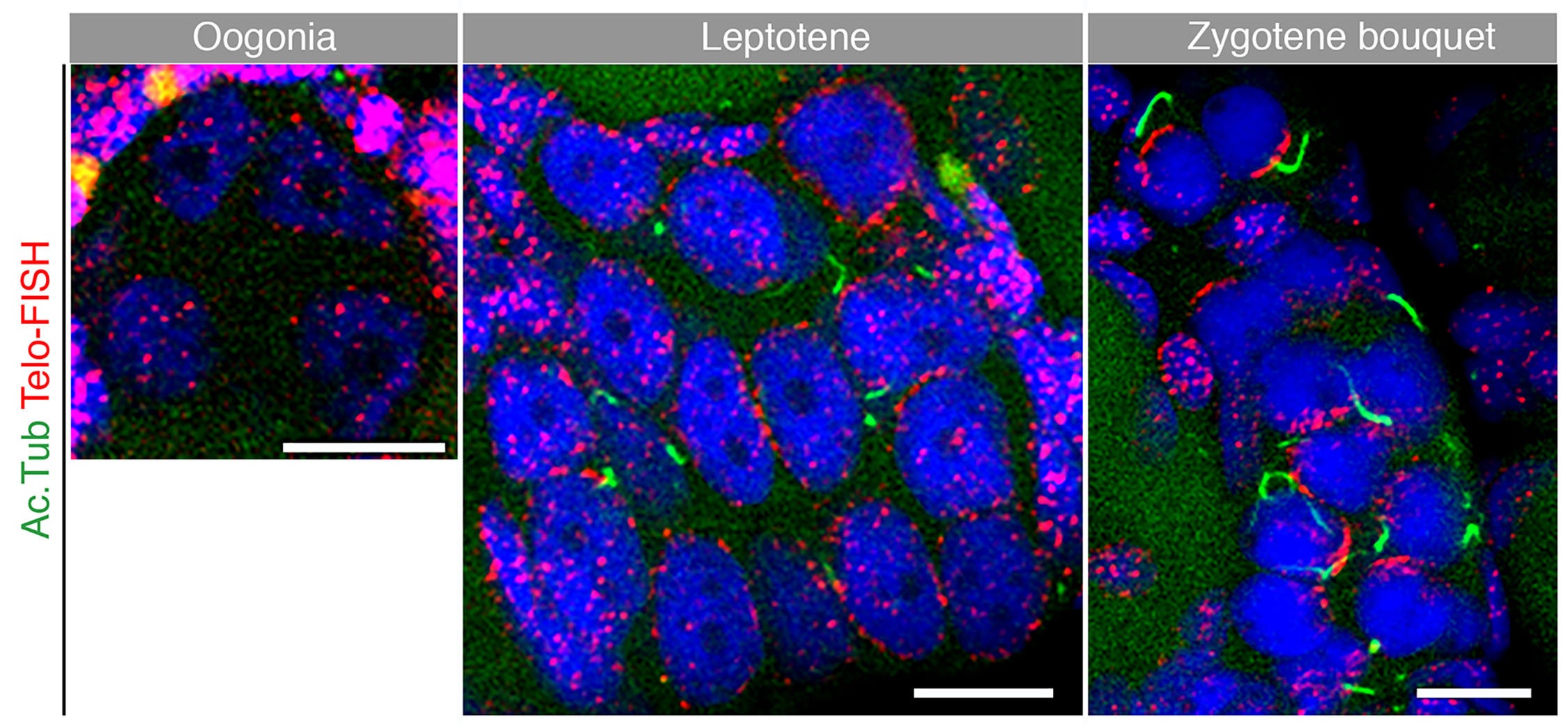

Supplement: S6 Fig — Telo-FISH staining (red) confirms the stage specificity of the acetylated tubulin cables (green) in the nest (costained with DAPI, blue). Premeiotic oogonia with intranuclear scattered telomeres (left) show no acetylated tubulin cables. Leptotene oocytes with telomeres loaded radially on the NE show some cables (center). Zygotene oocytes with tightly clustered telomeres on the NE show more elaborated cables associated with them. n = 6 ovaries. Scale bar: 10 μm. (TIF) [file pbio.1002335.s007.tif]
